# Systems genetics approaches model the heritable architecture of polyendocrine metabolic ovarian syndrome

**DOI:** 10.1172/JCI198215

**Published:** 2026-08-06

**Authors:** Christy M. Nguyen, Leandro M. Velez, Youngseo Cheon, Cimone L. Jackson, Casey D. Johnson, Ian Tamburini, Mingqi Zhou, Erik Alvstad, Isoo Yoon, Farheen Dustagheer, Marie Li, Tvisha Gujjarlapudi, Kaitlene Ofilan, Neha Mishra, Evan G. Williams, Danica Kwan, Carlos H. Viesi, Naveena Ujagar, David G. Ashbrook, Alistair Senior, Marin E. Nelson, Nicholas R. Pannunzio, Selma Masri, Evgeny Z. Kvon, Grant MacGregor, Cholsoon Jang, Vittorio Sebastiano, Minji Byun, Changrui Xiao, Alexander S. Kauffman, Robert W. Williams, David E. James, Ivan Marazzi, Dequina Nicholas, Marcus Seldin

**Affiliations:** 1 Department of Biological Chemistry and; 2 Center for Epigenetics and Metabolism, University of California, Irvine, Irvine, California, USA.; 3 Luxembourg Centre for Systems Biomedicine, University of Luxembourg, Belval, Luxembourg.; 4 Department of Molecular Biology and Biochemistry, Charlie Dunlop School of Biological Sciences, University of California, Irvine, Irvine, California, USA.; 5 Institute for Neuroscience and Cardiovascular Research, The University of Edinburgh, Edinburgh, United Kingdom.; 6 School of Life and Environmental Sciences and; 7 Charles Perkins Centre, University of Sydney, Camperdown, New South Wales, Australia.; 8 Chao Family Comprehensive Cancer Center, University of California, Irvine, Irvine, California, USA.; 9 Department of Developmental and Cell Biology, Charlie Dunlop School of Biological Sciences,; 10 Department of Microbiology and Molecular Genetics, School of Medicine, and; 11 Department of Neurology, University of California, Irvine, Orange, California, USA.; 12 Department of Obstetrics, Gynecology and Reproductive Sciences, University of California, San Diego, La Jolla, California, USA.; 13 Department of Genetics, Genomics and Informatics, University of Tennessee Health Science Center, Memphis, Tennessee, USA.

**Keywords:** Endocrinology, Metabolism, Reproductive biology, Complex traits, Population genetics, Sex hormones

## Abstract

Polyendocrine metabolic ovarian syndrome (PMOS), formerly known as polycystic ovary syndrome (PCOS), is the most common endocrine disorder in women and is closely associated with complex diseases such as cardiovascular disease and type 2 diabetes. However, the mechanistic links between PMOS and its comorbidities remain poorly understood. Here, we present an integrative systems genetics platform that leverages genetic diversity in both mice and humans to dissect the drivers of PMOS and its associated complications. This framework uncovered conserved genetic and environmental factors underlying PMOS, identified susceptible cell types and organs, and elucidated mechanisms linking PMOS to subsequent pathologies. For instance, we showed that increased ovarian area contributes to both PMOS susceptibility and ovarian cancer progression, while specific ovary–heart signaling circuits modulate cardiac function with aging. We further identified ovarian SF3B1-mediated alternative splicing as a key mechanistic link between PMOS and metabolic traits. Pharmacologic inhibition of SF3B1 in mice reduced circulating testosterone, insulin, and glucose levels as well as fat mass expansion. Transcriptomics analysis of ovaries from mice and experiments using human cell lines localized these effects to exon skipping events in granulosa cells. Together, this study offers a mechanistic framework for modeling the diversity of PMOS pathologies and uncovers SF3B1-mediated splicing as a link between ovary function and systemic metabolism.

## Abstract 2

We use inter-individual variation to define how PCOS predisposes to complex disease and develop a new theraputic strategy to treat these interactions

## Introduction

Polyendocrine metabolic ovarian syndrome (PMOS), formerly known as polycystic ovary syndrome (PCOS), is the most prevalent endocrinopathy in women, with an estimated occurrence of approximately 12% among those of reproductive age (1, 2). Characterized by a heterogeneous etiology encompassing reproductive, metabolic, and psychological features, PMOS poses major challenges in diagnosis and treatment (3, 4). The complex pathophysiology of PMOS arises from an interplay between genetic and environmental factors (5); however, comprehensive studies aimed at elucidating the genetic architecture of the syndrome remain highly limited. Beyond the comparatively well-documented implications for fertility (3), PMOS is intricately linked to a spectrum of cardiometabolic disorders, including insulin resistance (6), obesity (7), cancer (8), cardiovascular disease (9), and type 2 diabetes (10). Despite these observations, the underlying mechanisms linking PMOS with cardiometabolic disease and associated risk factors remain unknown. Although rodent models of PMOS have been developed to experimentally dissect disease mechanisms (11, 12), these studies typically rely on a single genetic background, limiting their generalizability and translatability in mimicking the spectrum of phenotypes observed in humans. In parallel, human genome-wide association studies (GWAS) (13–21) have implicated several genetic loci in PMOS risk but face limitations in pinpointing causal mechanisms related to effect size of associations, inconsistent diagnostic criteria, elusive cells and organs mediating susceptibility, and inability to accurately account for environmental influences.

Consistent with the lack of knowledge linking PMOS to associated comorbidities, current treatment strategies remain fragmented. Clinical regimens either focus on restoring fertility or managing complications related to conditions such as obesity or type 2 diabetes (3, 22). Although ovulation induction therapies and lifestyle modifications may address reproductive symptoms (23) or metabolic dysfunction (24), respectively, no unified approach exists that simultaneously targets the full spectrum of PMOS-related pathophysiologies (3). This includes the interconnected endocrine, ovarian, and cardiometabolic abnormalities that collectively define the syndrome. As a result, many women receive piecemeal care that fails to address the root causes or long-term health risks of PMOS, underscoring the urgent need for integrated therapeutic strategies that account for the complex and systemic nature of the disease. In the present study, we aimed to bridge these gaps by developing a new genetic reference panel for PMOS in highly diverse inbred mouse strains and matched placebo controls, integrated with human variation in cardiometabolic outcomes. By combining a new genetic reference panel with available human clinical and molecular data, we propose reproducible models of human PMOS subtypes as well as potential mechanisms of ovarian cancer susceptibility and ovary-heart communication. As a proof of principle, we identified RNA splicing via SF3B1 as a mechanistic bridge between ovarian function and metabolic outcomes. Treatment with a specific inhibitor of SF3B1 (H3B-8800) (25, 26) in adult mice was sufficient to rescue many PMOS-induced systemic features, improving key metabolic and hormonal traits.

## Results

### Diversity of genetic responses to PMOS in mice and potential models of human PMOS subtypes.

**Figure 1:**
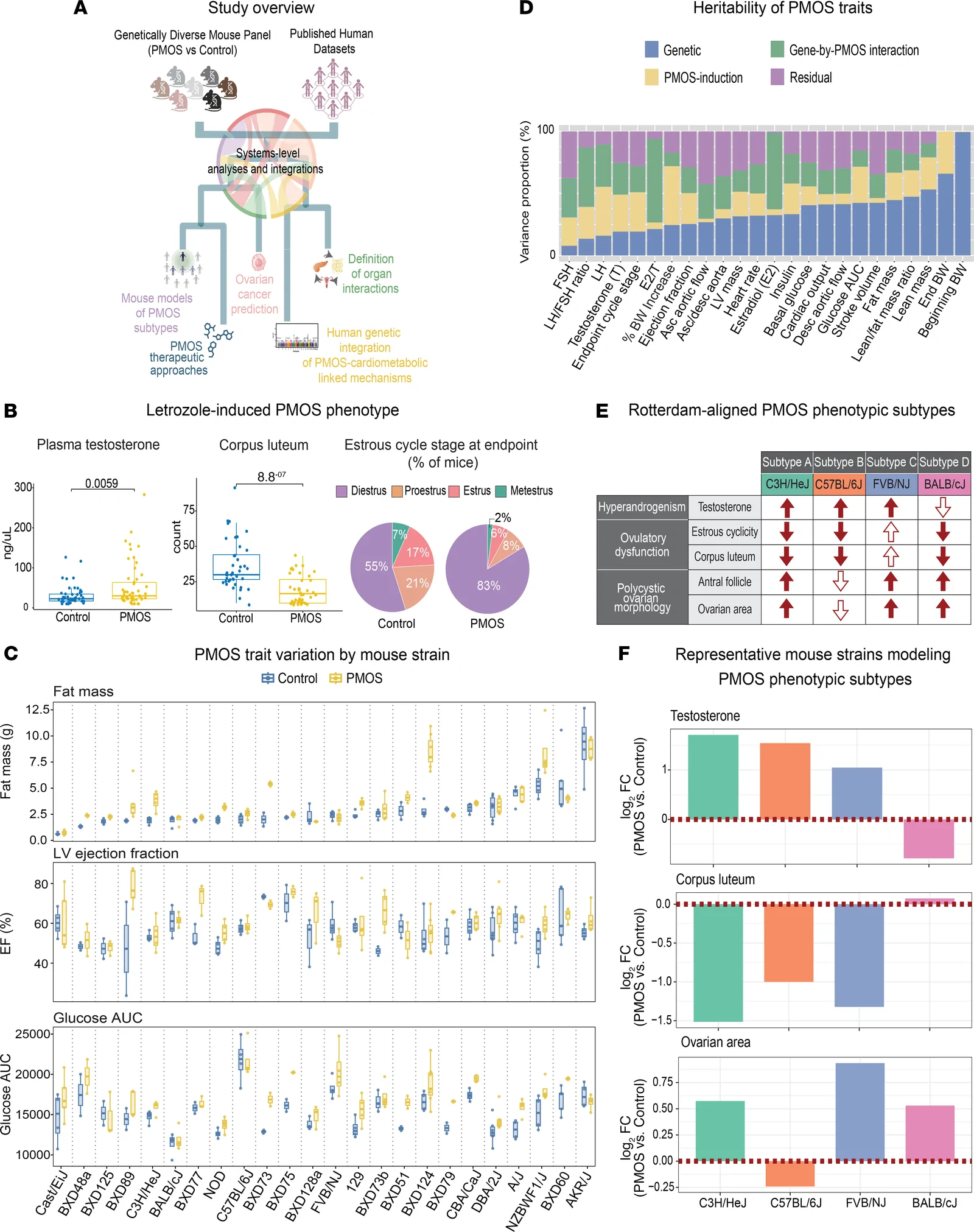
Systems genetics approach for modeling PMOS phenotypic diversity. (**A**) Study overview illustrating integration of genetically diverse mouse models with human datasets to uncover molecular drivers of PMOS phenotypic diversity (42–48, 54, 64, 65). (**B**) Plasma testosterone, corpus luteum number, and percentage of mice in each estrous cycle stage at endpoint in control and letrozole-induced PMOS mice (*n* = 50–53 mice per group). *P* values were determined by unpaired 2-tailed Student’s *t* test. (**C**) Phenotypic variation across genetically diverse mouse strains, including fat mass, left ventricular ejection fraction, and glucose clearance, measured as area under the curve, in control and PMOS mice (*n* = 3–4 mice per strain per group). In **B** and **C**, points represent individual mice; boxes show the median and interquartile range, with whiskers extending to 1.5× the interquartile range. (**D**) Percentage of variance in PMOS-associated traits attributed to genetic, PMOS induction, gene-by-PMOS interaction, and residual components. (**E**) Mapping of mouse phenotypic traits and their direction across strains aligned to human Rotterdam criteria to define corresponding mouse models of PMOS subtypes. Arrows indicate direction of change in PMOS relative to control mice; filled arrows indicate traits included in defining each subtype, and open arrows indicate traits not included. (**F**) Representative mouse strains modeling Rotterdam-aligned PMOS subtypes, shown by log_2_ fold change in PMOS relative to control mice for circulating testosterone, corpus luteum number, and ovarian area. Dashed lines indicate no change.

To elucidate the genetic underpinnings of PMOS and its interactions with reproductive and cardiometabolic traits, we modeled PMOS in 233 diverse inbred female mice alongside genetic replicates, achieved through aromatase inhibition (27). Molecular, biochemical, and physiologic interrogation of mouse data was integrated with human cohort studies to pinpoint conserved mechanisms bridging PMOS to complex diseases (Figure 1A). This model closely mimicked core features of the disease across strains (Figure 1B and Supplemental Table 1; supplemental material available online with this article; https://doi.org/10.1172/JCI198215DS1). Moreover, the range of physiologic responses to PMOS in the context of genetic diversity was markedly greater than responses to PMOS induction in any single genetic background (Figure 1C and Supplemental Figure 1), highlighting the importance of surveying complex phenotypes across diverse genetic backgrounds. To quantify the contributions of genetics, environment, and their interactions to PMOS-induced outcomes, we used linear mixed models to partition the variance of selected traits into strain (genetic heritability; h2), environmentally induced PMOS effect, and strain-by-PMOS effects, akin to gene-by-environment interactions (Figure 1D) (28–30). On average, most traits showed a strong genetic contribution (30%–60%), but we also observed substantial variance attributable to gene-by-PMOS interactions (Figure 1D), underscoring the complex set of interactions facilitating disease development and heterogeneity. We note that several traits exhibited high levels of residual effects where no immediate source of variation could be attributed. For example, follicle-stimulating hormone (FSH) levels showed approximately 30% of residual variation. For these outcomes, caution should be used when interpreting in the context of this resource, as potential confounding factors such as food intake or energy expenditure could be influencing the levels of variation.

PMOS is a highly heterogeneous disorder, with subtypes defined under the Rotterdam criteria by combinations of hyperandrogenism, ovulatory dysfunction, and polycystic ovarian morphology (PCOM) (3). To model this heterogeneity, we classified mouse strains according to the directionality of PMOS-associated traits relative to controls. Hyperandrogenism was defined by increased circulating testosterone, ovulatory dysfunction by disrupted estrous cyclicity and decreased corpus luteum number, and PCOM by elevated antral follicle counts and increased ovarian area. Stratifying mouse strains by the presence or absence of these features revealed 4 phenotypic groups that parallel clinical PMOS subtypes (A–D) (Figure 1E). We identified different mouse strains that model distinct combinations of PMOS-associated traits, such as hyperandrogenism- versus PCOM-dominant mouse models. For example, our stratification revealed that the C3H/HeJ strain most closely represented subtype A (hyperandrogenism and ovulatory dysfunction), exhibiting elevated testosterone, arrested estrous cyclicity, and enlarged ovarian area (Figure 1F). In contrast, the BALB/cJ strain most closely reflected subtype D (ovulatory dysfunction and PCOM), characterized by pronounced estrous cycle arrest and increased ovarian area but comparatively lower circulating testosterone than observed in the hyperandrogenic subtype A model (Figure 1F).

### Integration of ovarian histology and transcriptomics proposes mechanisms predisposing to cancer severity.

**Figure 2:**
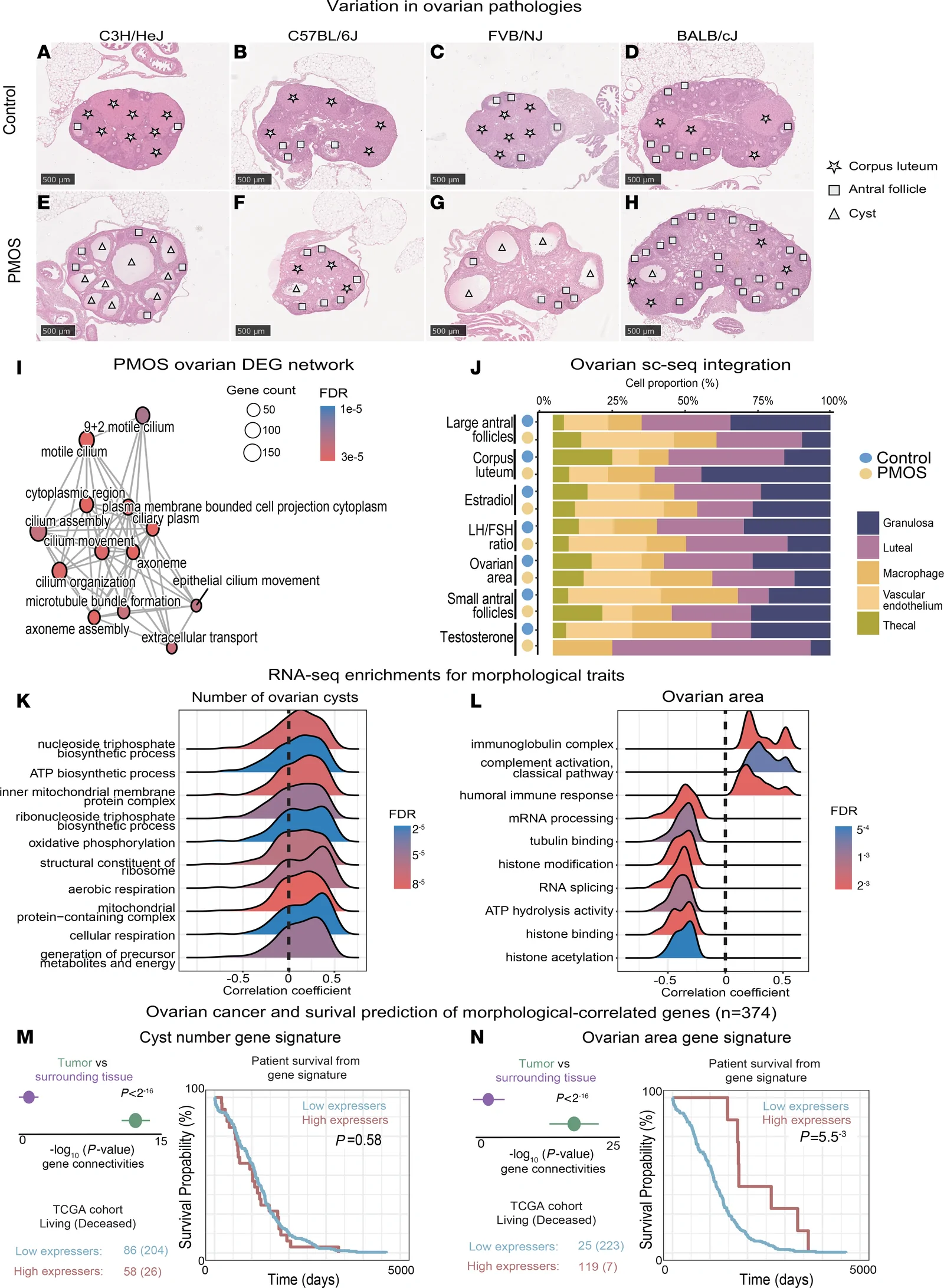
Genetic variation in ovarian morphology defines distinct cellular and molecular states with clinical relevance. (**A**–**H**) Representative ovarian histology across 4 mouse strains, C3H/HeJ, C57BL/6J, FVB/NJ, and BALB/cJ, under control (**A**–**D**) or letrozole-induced PMOS (**E**–**H**) conditions. Stars, squares, and triangles indicate corpora lutea, antral follicles, and cysts, respectively. Scale bars: 500 μm. (**I**) Gene set enrichment analysis network of pathways enriched from differentially expressed ovarian genes in PMOS versus control mice. Edges represent shared genes between pathways. Node size indicates gene count, and node color indicates FDR. (**J**) Cell-type distribution of trait-associated genes in control and PMOS mice across ovarian morphological and hormonal traits, based on ovarian cell–type annotation. Bar colors indicate granulosa, luteal, macrophage, vascular endothelial, and thecal cell–specific transcriptional signatures. (**K** and **L**) Correlation coefficients of genes within the top enriched pathways associated with ovarian cyst number (**K**) or total ovarian area (**L**) across strains. Colors indicate FDR for each pathway, calculated from the enrichment statistics. (**M** and **N**) Gene expression signatures derived from positively correlated genes in **K** and **L** were evaluated in TCGA ovarian cancer samples. Images on the left show mean expression in tumor versus surrounding tissue and counts of deceased individuals used for survival analysis. Images on the right show Kaplan-Meier curves for patient survival, stratified by high and low expression relative to the population mean, using genes correlated with ovarian cyst number (**M**) or ovarian area (**N**). Tumor versus surrounding tissue *P* values were calculated using the Wilcoxon test. Survival *P* values were calculated using the log-rank test.

Ovarian dysfunction is a pivotal feature of PMOS and resulting comorbidities in humans. Similar to cardiometabolic and hormonal changes, ovarian pathologies differed based on the genetic background (Figure 2, A–H). For instance, the C3H/HeJ strain (similar to subtype A in humans) showed extensive ovarian cyst formation and an almost complete loss of corpora lutea following letrozole treatment (Figure 2, A and E). In contrast, BALB/cJ mice showed similar numbers of corpora lutea and limited cyst formation between control and letrozole-treated groups; however, they exhibited pronounced expansion of ovarian area following PMOS induction (Figure 2, D and H). In contrast, BALB/cJ mice showed similar corpus luteum and limited cyst formation; however, they also had pronounced expansion of ovarian area following PMOS induction (Figure 2H). Similar to variation in cardiometabolic responses, these combinations of histologic phenotypes have never been modeled experimentally across diversity, highlighting the range of histologic variation that can be used to model PMOS. Ovarian RNA-seq of all strains showed that pathways related to cilium movement were the most reduced between control and PMOS mice (Figure 2I and Supplemental Table 2), consistent with disrupted estrous cyclicity (31, 32). Bulk ovarian sequencing and histological data were integrated with single-cell ovarian transcriptomics (33) to define the cell-specific changes associated with PMOS and related outcomes. Variation in expression of cell populations such as granulosa cells, luteal cells, thecal cells, macrophages and endothelial cells revealed distinct correlation patterns with PMOS traits (Figure 2J and Supplemental Table 3). Most notably, testosterone-associated genes shifted from predominantly vascular endothelial and macrophage signatures in controls to a luteal-cell signature in PMOS mice.

Broadly, counting cysts or follicles has been performed in mice, but its clinical specificity is limited owing to its high prevalence in healthy and adolescent populations and variability with imaging methodology (34). In contrast, ovarian size and stromal expansion, reflected by ovarian area or stromal-to-ovarian area ratios, are independently incorporated into diagnostic criteria and more closely associated with androgen excess and underlying ovarian dysfunction, supporting their relevance as physiologically meaningful markers (35). Furthermore, features of PMOS such as elevated androgen levels can predispose women to ovarian cancer development and serve as markers of patient survival (8, 36–38); however, the number of cysts detected has shown limited predictive capacity (39–41). To dissect these relationships, we developed gene signatures corresponding to distinct cellular functions and tested their ability to predict ovarian cancer development in The Cancer Genome Atlas (TCGA) program (42, 43). Specifically, we assigned gene expression scores based on human orthologs of mouse genes correlated with either number of ovarian cysts or total ovarian area, which were enriched in respiration/ATP synthesis (Figure 2K) or immune/RNA processing pathways (Figure 2L), respectively. While both gene signatures were elevated in ovarian tumors compared with surrounding tissue, only the orthologs correlated with ovarian area measures predicted patient survival (Figure 2, M and N). Notably, this new gene signature focused on ovarian area expansion outperformed several existing models developed explicitly for ovarian cancer survival prediction in TCGA (Supplemental Table 4). These findings demonstrate the range of genetic responses to PMOS in ovarian function and show that ovarian area changes in PMOS serve as predictors of ovarian cancer severity.

### PMOS shifts network structure of adipose estradiol signaling in mice and ovary-heart communication.

**Figure 3:**
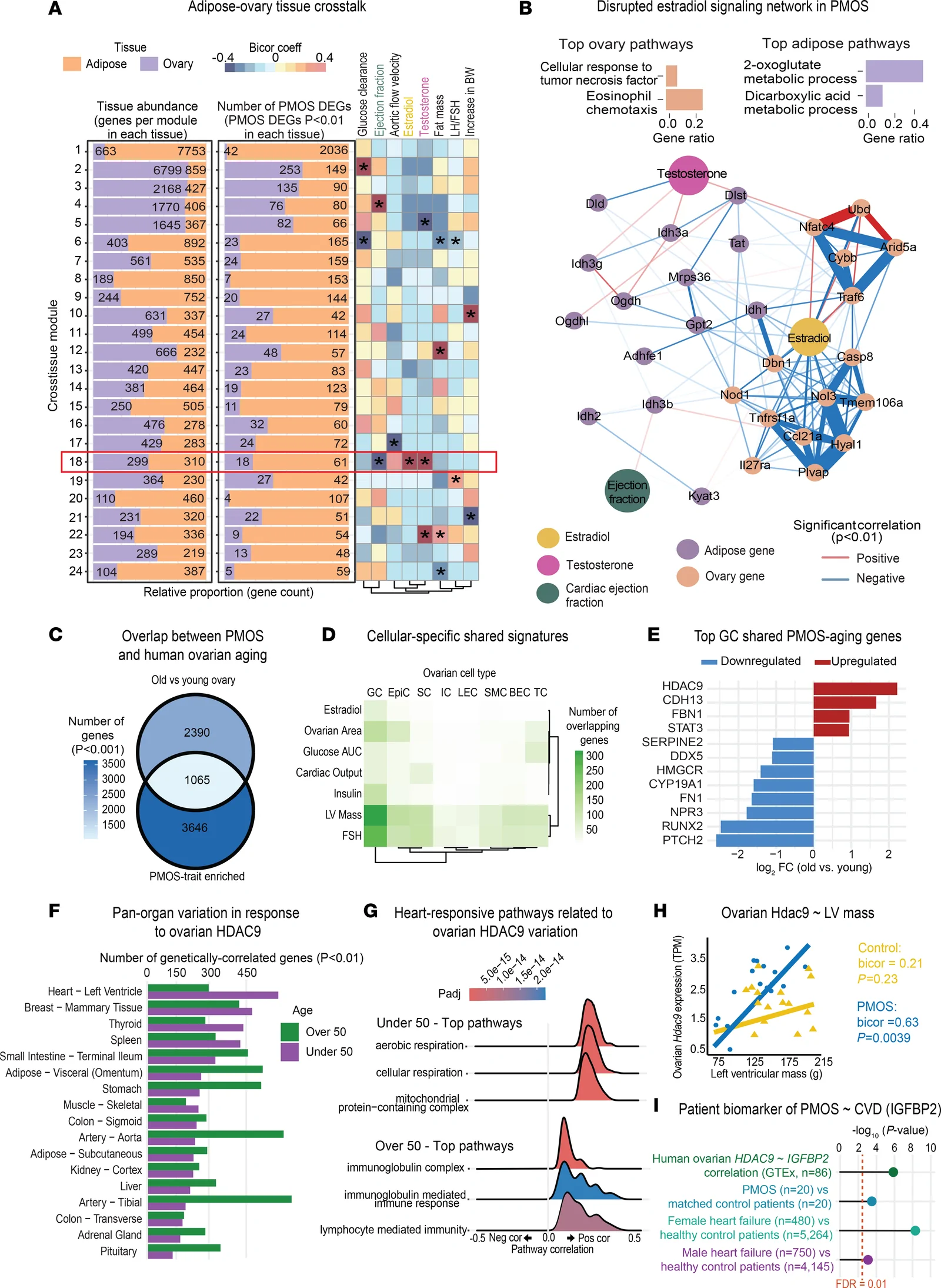
Ovary–peripheral organ crosstalk links reproductive and cardiometabolic phenotypes in PMOS. (**A**) Weighted gene coexpression network analysis of ovarian and adipose gene expression in PMOS mice. The image on the left indicates the tissue composition of each module, the center image shows the number of PMOS-associated differentially expressed genes in each tissue, and the image on the right shows correlations between module eigengenes and hormonal and cardiometabolic traits. Asterisks indicate statistically significant associations. Module 18 is highlighted based on correlations with testosterone, estradiol, and left ventricular ejection fraction. (**B**) Undirected network of module 18 genes. Node color indicates ovary-derived genes, adipose-derived genes, estradiol, testosterone, or cardiac ejection fraction. Edges represent statistically significant correlations, with red and blue indicating positive and negative correlations, respectively. Edge weight corresponds to correlation strength. Enriched ovarian and adipose pathways are shown by gene ratio. (**C**) Overlap between PMOS trait-correlated genes and aging-associated genes in human ovary. (**D**) Heatmap showing overlap between PMOS trait-associated genes and ovarian cell–type signatures. (**E**) Top overlapping granulosa cell genes shown by log_2_ fold change in aged versus young ovary. (**F**) Number of genes correlated with ovarian *HDAC9* expression across tissues, stratified by age. (**G**) Gene set enrichment analysis of left ventricular pathways associated with ovarian *HDAC9* expression by age group. (**H**) Ovarian *Hdac9* expression plotted against left ventricular (LV) mass in control and PMOS mice. (**I**) Association of ovarian *HDAC9* with *IGFBP2* expression in human ovary and circulating IGFBP2 levels in PMOS and heart failure cohorts. Correlations were calculated using biweight midcorrelation. Differential expression was assessed using limma with FDR adjustment where indicated. Associations in **A**, **B**, **F**, **H**, and the ovarian correlation in **I** were calculated using biweight midcorrelation, with *P* values determined using bicorAndPvalue. In **C**, the Venn diagram shows the descriptive intersection of genes meeting *P* < 0.001 in the respective PMOS trait-correlation and human ovarian-aging analyses. Gene set enrichment analysis in **G** was performed using clusterProfiler, with adjusted *P* values shown. Differential protein abundance in **I** was assessed using limma with FDR adjustment.

Given the close anatomical proximity and critical role of adipose tissue in maintaining cardiometabolic homeostasis, we sequenced gonadal fat from the cohort and compared its gene expression directly with that of the ovaries. Genetic variation in adipose tissue revealed broad changes related to lipid processing in PMOS (Supplemental Figure 2 and Supplemental Table 5) and integration of single-nuclear sequencing (44–46), and genome-wide association colocalization analyses (47, 48) highlighted conserved shared mechanisms linking PMOS to heart function and glucose metabolism (Supplemental Figure 3). Network topology approaches (49–51) pinpointed a specific circuit of ovary-adipose crosstalk that is selectively disrupted in PMOS (Figure 3A). These analyses suggest that circulating estradiol coordinates ovarian inflammation with adipose ketone body utilization, potentially enabling the hormone to maintain cardiac ejection fraction (Figure 3B). However, confirmation of this mechanism would require direct experimental evidence testing estradiol actions in relevant systems.

PMOS susceptibility, hormonal balance, and coordinated ovarian functions change substantially with age (52, 53). To dissect these relationships across species, we integrated trait-associated ovarian gene-expression patterns from the present PMOS mouse cohort with published single-nucleus RNA-sequencing profiles of human ovarian aging (54). These analyses showed distinct ovarian changes in PMOS and aging (Figure 3C and Supplemental Figure 4), with shared features occurring in granulosa cells (Figure 3D). We filtered human granulosa cell–specific aging DEGs to identify those shared with mouse orthologs that were both differentially expressed in PMOS and correlated with at least 3 phenotypes. This filtering revealed established markers of ovarian homeostasis and metabolism (55, 56) as well as new candidate drivers (Figure 3E). For example, ovarian *HDAC9* was upregulated in the granulosa cells of older individuals (Figure 3E) and exhibited selective cross-organ enrichments (51, 57–60) with heart processes during aging (Figure 3F). Specifically, in individuals below 50 years of age, heart pathways associated with ovarian *HDAC9* reflected increased mitochondrial capacity, whereas in older individuals, the top pathways shifted toward an immune response (Figure 3G). Consistent with patterns observed in human genetic analyses, ovarian *Hdac9* expression correlated with left ventricular mass specifically in PMOS mice (Figure 3H). Granulosa cell HDAC9 has been shown to impact functions such as inflammation (61) and cell state (62, 63). Our analyses suggest its induction during aging may serve as a mechanistic link between ovarian status and heart function.

Based on these results, we next wanted to identify accessible biomarkers of shared PMOS and cardiovascular disease. By comparing proteomic measures between age and BMI-matched PMOS-diagnosed and healthy women (64), then following these signatures across sex-stratified analyses of heart failure (65) cases and controls, we identified the circulating protein IGFBP2 as a predictor of shared PMOS and left ventricular expansion (Figure 3I). We note that plasma levels of IGFBP2 were strongly linked to heart failure in women, with a more modest association observed in men (Figure 3I). Collectively, these results provide a framework to pinpoint the organ communication networks that are globally disrupted in PMOS and aging as well as specific factors that underlie signaling mechanisms between tissue processes.

### Inhibition of ovarian SF3B1 is sufficient to rescue metabolic and hormonal perturbations following PMOS induction.

**Figure 4:**
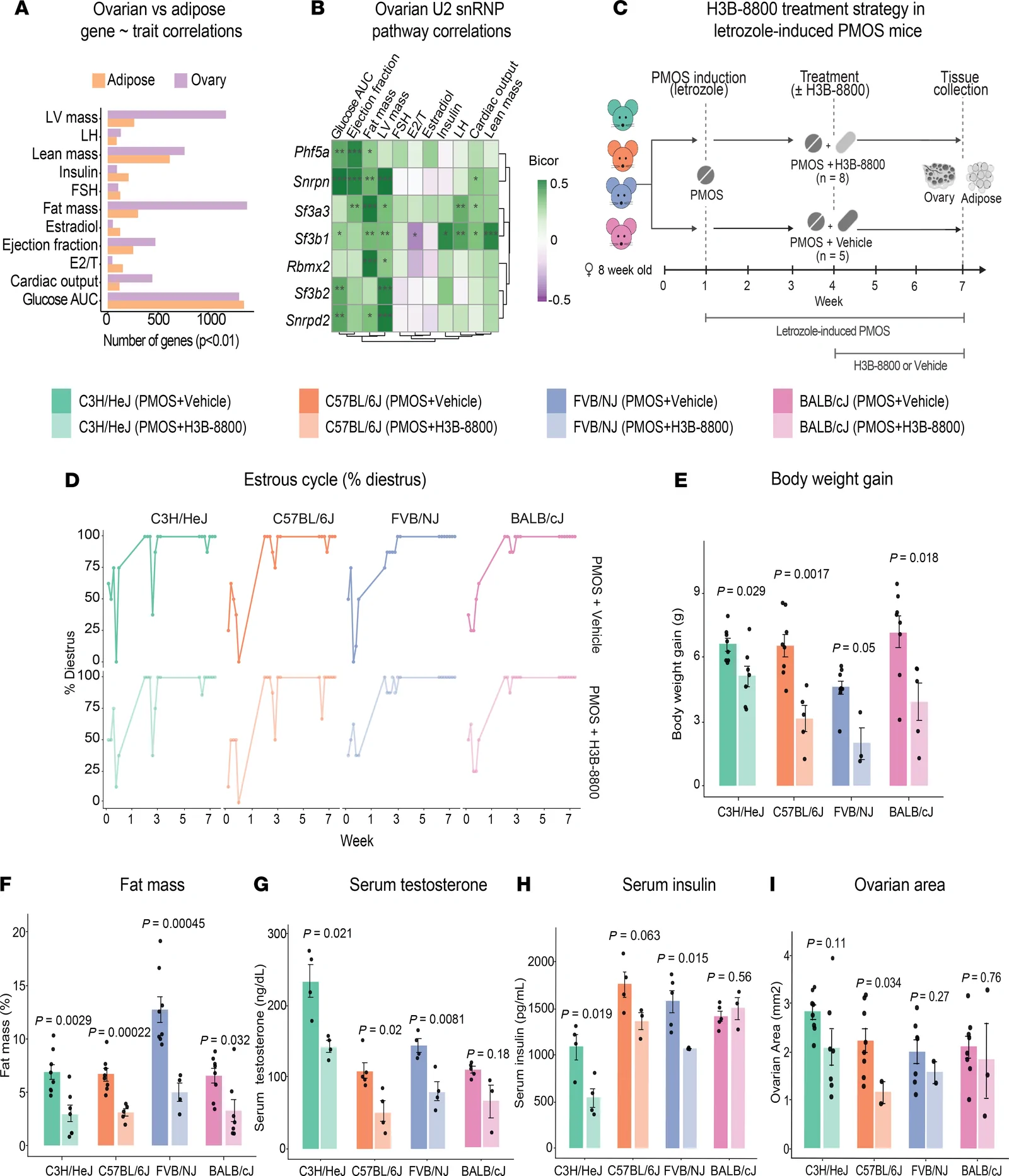
H3B-8800 selectively improves hormonal and metabolic phenotypes across genetically diverse mouse models of PMOS. (**A**) Number of ovarian (purple) and adipose (orange) genes significantly correlated (*P* < 0.01) with systemic traits across PMOS mice. (**B**) Heatmap of biweight midcorrelation (bicor) coefficients between expression of ovarian U2 small nuclear ribonucleoprotein (snRNP) spliceosomal components and systemic traits. Color scale represents correlation strength, and asterisks indicate significance (*P < 0.05, **P < 0.01, ***P < 0.001). (**C**) Experimental design of H3B-8800 treatment in letrozole-induced PMOS mice across genetically diverse strains. (**D**) Estrous cyclicity assessed as the percentage of days each mouse spent in diestrus across the study period in PMOS mice treated with vehicle or H3B-8800 (*n* = 4–8 mice per strain per treatment group). (**E**–**I**) Body weight gain (**E**), fat mass (**F**), serum testosterone (**G**), serum insulin (**H**), and ovarian area (**I**) in PMOS mice treated with vehicle or H3B-8800 across strains (*n* = 2–8 mice per strain per treatment group). Vehicle and H3B-8800 comparisons were performed 3 weeks after initiation of vehicle or H3B-8800 treatment, following 3 weeks of letrozole-induced PMOS induction. Data are shown as mean ± SEM. *P* values were determined by unpaired 2-tailed Student’s *t* test within each strain.

Our next goal was to leverage this systems genetics platform to identify actionable mechanisms with therapeutic potential for addressing both reproductive and metabolic dysfunctions in PMOS. Global gene correlation analyses comparing reproductive, hormonal, and cardiometabolic traits showed stronger associations with ovarian gene expression than with adipose tissue in PMOS conditions (Figure 4A). Specifically, we observed enrichment of genes involved in the U2 small nuclear ribonucleoprotein complex of the spliceosome (66). Genetically driven variation in ovarian splicing factor 3b subunit 1 (*Sf3b1*) expression was correlated with a spectrum of PMOS and metabolic traits (Figure 4B).

A selective inhibitor of SF3B1 (H3B-8800) was recently developed to target spliceosome-mutant cancers (25, 67, 68). This small molecule has completed phase I clinical trials (26); however, its potential beyond hematologic malignancy remains unknown. Therefore, we induced PMOS via aromatase inhibition in 4 mouse strains representing distinct human subtypes (Figure 1, E and F) and tested how H3B-8800 alters reproductive and metabolic phenotypes (Figure 4C). Consistent with studies in C57BL/6J mice (27), letrozole disrupted estrous cyclicity as early as 2–3 weeks after treatment and promoted weight gain (Figure 4E). A time-course analysis of letrozole response of these strains allowed us to sequentially order changes in phenotypes observed. Here, changes in testosterone appeared first, followed by disrupted estrous cyclicity, and then changes in body weight and glucose levels (Supplemental Figure 5). These data demonstrate that the hormonal and reproductive changes are upstream of cardiometabolic outputs in the context of this model. Among all 4 genetic backgrounds, H3B-8800 treatment decreased body weight expansion, specifically by reducing fat mass (Figure 4, E and F). H3B-8800 administration under continued letrozole exposure did not restore estrous cyclicity, and mice remained persistently arrested in diestrus through the completion of the study (Figure 4D). Other PMOS-related outcomes showed genetic background-dependent responses to H3B-8800 treatment. For example, serum testosterone and insulin were reduced in C57BL/6J, C3H/HeJ, and FVB/NJ mice, whereas BALB/cJ mice were resistant to these effects (Figure 4, G and H). In addition, ovarian area was reduced via H3B-8800 in C57BL/6J mice selectively (Figure 4I). Many other reproductive and metabolic phenotypes showed variable responses to H3B-8800 following PMOS induction (Supplemental Figures 5 and 6), suggesting distinct reprogramming mechanisms required for differing PMOS subtypes. Beyond SF3B1 inhibition, network-based drug prioritization approaches (69, 70) identified multiple drug classes, both established and underexplored, enriched across cardiometabolic traits, including antidiabetic agents, PPAR agonists, and hormone signaling pathways (Supplemental Figure 7).

**Figure 5:**
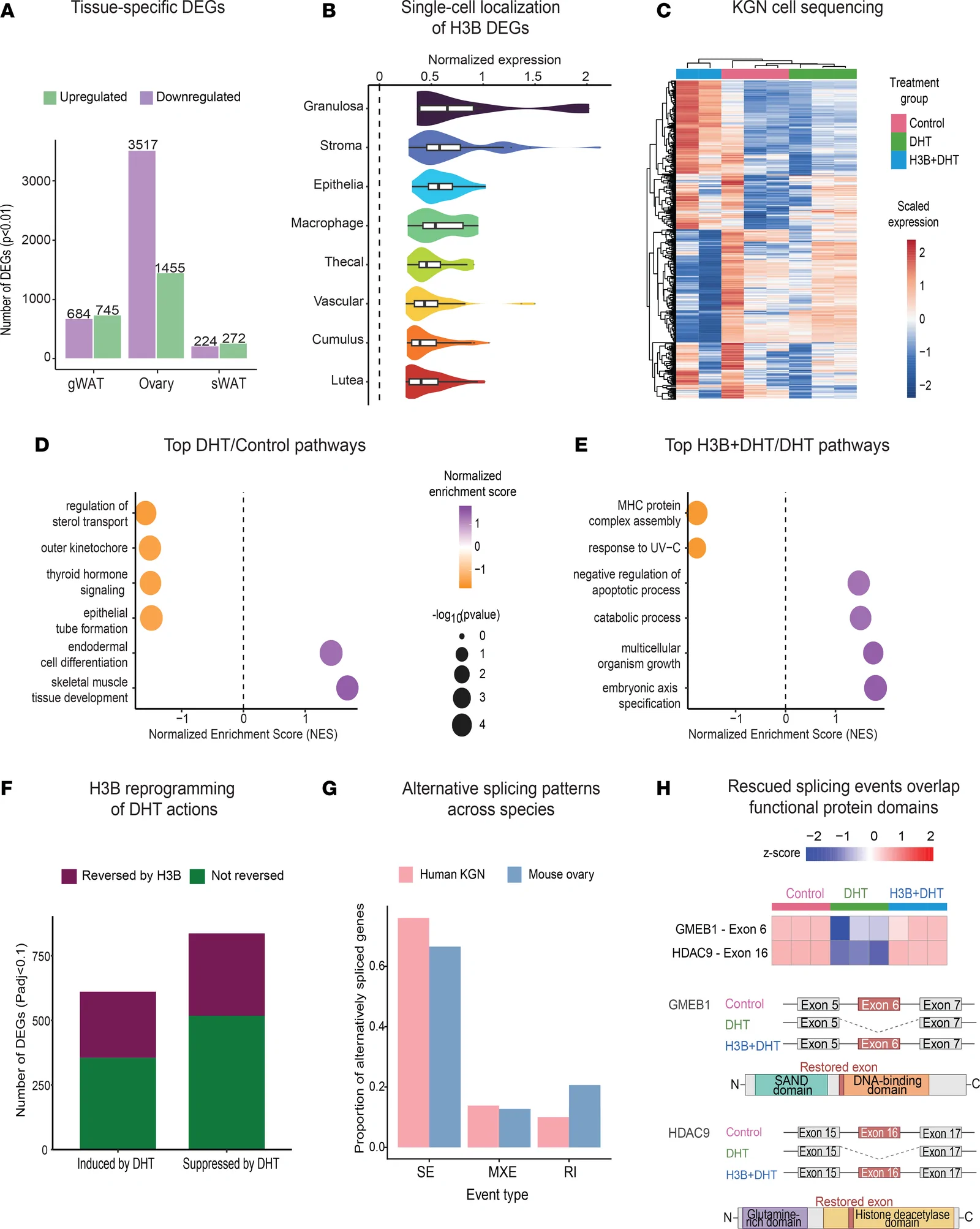
H3B-8800 reprograms hyperandrogenism-induced dysregulation of granulosa cell transcriptional and splicing programs. (**A**) Number of upregulated and downregulated genes across ovary, gonadal white adipose tissue (gWAT), and subcutaneous white adipose tissue (sWAT) in PMOS mice treated with H3B-8800 or vehicle. (**B**) Single-cell expression of H3B-8800–associated differentially expressed genes across ovarian cell types. (**C**) Heatmap of row-scaled gene expression in human granulosa KGN cells across control, DHT, and H3B-8800+DHT conditions (*n* = 3 per treatment group). Color indicates relative expression. (**D** and **E**) Gene set enrichment analysis of differentially expressed genes showing normalized enrichment scores for selected pathways in DHT-treated versus control KGN cells (**D**) and H3B-8800+DHT–treated versus DHT-treated KGN cells (**E**). Point color indicates direction of enrichment, and point size indicates –log10(P value). (**F**) Number of DHT-induced or DHT-suppressed differentially expressed genes, categorized by whether expression was reversed by H3B-8800 treatment. (**G**) Distribution of the top alternative splicing event types in human KGN cells and mouse ovarian tissue, including skipped exon (SE), mutually exclusive exon (MXE), and retained intron (RI) events. (**H**) Heatmap showing percentage spliced in values, scaled as *z*-scores, for representative alternative splicing events in *GMEB1* and *HDAC9* across control, DHT, and H3B-8800+DHT conditions (*n* = 3 per treatment group). Schematics depict exon structure and protein domains, showing restoration of alternative exon usage within functionally relevant regions. Differential expression was assessed using limma, and pathway enrichment was assessed by gene set enrichment analysis.

Given that inhibition of SF3B1 improved hormonal and metabolic traits in vivo, we next wanted to (a) pinpoint the organs and mechanisms by which H3B-8800 was acting and (b) test the degree of conservation to humans. Despite the subcutaneous route of administration, H3B-8800 treatment altered gene expression profiles selectively in the ovary (Figure 5A and Supplemental Table 6). Among DEGs, we observed minimal overlap between ovary and fat pads (Supplemental Figure 8). Single-cell localization of H3B-8800–associated DEGs using a mouse ovarian transcriptomic atlas (33) showed that these transcriptional changes were most strongly enriched in granulosa cells (Figure 5B). To test whether H3B-8800 acted directly on granulosa cells, we treated human KGN cells with dihydrotestosterone (DHT) to mimic androgen excess (71, 72) alongside H3B-8800, and performed RNA-seq. Androgen excess treatment showed strong shifts in gene expression, many of which were reversed via H3B-8800 treatment (Figure 5C and Supplemental Table 7). Specifically, DHT treatment suppressed steroid transport and thyroid hormone signaling processes, while inducing distinct developmental trajectories not observed in untreated granulosa cells (Figure 5D). The top expression pathways when DHT-treated KGN cells received H3B-8800 differed, as they showed a suppression in DNA damage and apoptosis (Figure 5E). We note that among the approximately 1,400 DEGs associated with DHT treatment, H3B-8800 reversed the expression changes of most (Figure 5F). Since H3B-8800 inhibits SF3B1 activity, we applied rMATS (73) to identify the types of transcriptional events associated with PMOS and H3B treatment. In both human KGN cells and mouse ovary, exon skipping appeared as the dominant process (Figure 5G). Further interrogation of skipped exon events helped to elucidate some potential mechanisms whereby H3B-8800 could be restoring key granulosa cell functional processes. For instance, the gene *GMEB1*, a cotranscriptional regulator of steroidogenic signaling (74) and glucocorticoid response (75), showed skipping of exon 6, a core component of the DNA-binding domain (76, 77) where exon retention was restored in H3B-8800 treatment (Figure 5H). Similar rescuing effects via H3B-8800 were observed for *HDAC9*, where DHT induced skipping of exon 16 within the deacetylase domain, an event that was restored by H3B-8800 (Figure 5H). These data show that granulosa cells could be an important source of H3B-8800 actions and provide some mechanistic links for the beneficial effects that are conserved in human cells.

## Discussion

Here, we present a mouse genetic reference panel to study the genetic basis for PMOS as well as corresponding analyses alongside human data to study mechanisms of coinciding cardiometabolic complications. By starting with a genetically diverse, replicable mouse population, we propose what we believe to be new model systems for subtypes of human PMOS. While mouse reference panels enable tighter control of environmental variables, no system is immune to the influence of confounding variables. Factors such as food intake, energy expenditure, and socialization behaviors, which have been shown to vary genetically in mice (78–80), will be important to consider when analyzing these data and others. We perform a variety of computational analyses using combined mouse and human genetic, transcriptomic, and phenotypic data to suggest mechanisms of PMOS that could relate to ovarian cancer, cardiovascular disease, and ovarian communication with peripheral organs. As proof of principle, we demonstrate that these data can be leveraged as a discovery tool to identify a new therapeutic strategy that rescues several hormonal and metabolic features of PMOS through SF3B1 inhibition. In sum, we emphasize how incorporating genetic diversity into PMOS models can assist in experimental systems and propose several analytical approaches for understanding whether mechanisms are conserved in humans.

The letrozole PMOS model used was originally created and validated by our coauthor, ASK, and his colleagues in a number of prior studies (27, 81–86). Thus, we used this model because it is well-validated and recapitulates key reproductive and metabolic features of PMOS observed in women. Compared with several prenatal, prepubertal, and postnatal androgen-exposure models, the letrozole model more consistently reproduces a broader combination of reproductive and metabolic PMOS-like traits (12). Some prenatal/prepubertal androgen models do not show consistently elevated luteinizing hormone (LH), and several postnatal androgen models show reduced or unchanged ovarian size/weight, which is less concordant with the enlarged polycystic ovary morphology often seen in women with PMOS (87–89). In this light, our hypothesis was that applying this model to a range of genetic variation in mice could help to bridge rodent models of PMOS with human disease. We and others previously showed that the PMOS phenotype is fully induced within approximately 2 weeks of continuous letrozole exposure and persists for at least 6 weeks. Longer periods beyond that have not been studied since the letrozole pellets are only viable for a 7-week duration. The timing of changes related to letrozole treatment are also important in interpreting the relationship between reproductive and metabolic outcomes. In this model, we observe elevated testosterone as the initial change observed, consistent with its canonical role in aromatase inhibition (27, 81–86). Following elevated testosterone, estrous cyclicity disruption occurred, which was subsequently followed by changes in metabolic parameters such as fat mass expansion and glucose levels. Based on these observations, it is critical to recognize that, in this model, androgen excess induces metabolic changes, rather than metabolic alterations causing androgen excess and its downstream consequences.

Clearly, changes in systemic metabolism have been shown to influence androgen levels and associated reproductive outcomes (90–92), a feature not captured in the present study. By integrating genetic, molecular, and physiological data across genetically diverse mouse strains, we identified SF3B1 as a central mediator linking local ovarian function to systemic metabolic outcomes in PMOS. Pharmacological inhibition of SF3B1 using H3B-8800 reduced circulating testosterone levels, weight gain, and gonadal fat mass in aromatase-inhibition PMOS mice. Under continuous letrozole exposure, H3B-8800 treatment produced modest ovarian structural changes and did not restore estrous cyclicity, suggesting that persistent aromatase blockade maintains reproductive axis suppression despite downstream metabolic rescue. Notably, most prior studies evaluating therapeutic rescue in letrozole-induced PMOS remove letrozole after approximately 3 weeks of treatment before assessing reproductive outcomes (93–95), limiting direct comparison to the present model, which evaluates the effects of H3B-8800 under sustained aromatase inhibition. Genetic models targeting direct regulators of the hypothalamic-pituitary-gonadal axis, such as gonadotropins (96), kisspeptin neurons (83, 97), and *Cyp11a1* (98), have restored reproductive function in PMOS models and may offer complementary strategies. Furthermore, the effect of SF3B1 inhibition on fertility outcomes such as pregnancy rates and live births remains to be determined. Future breeding studies will be necessary to evaluate conception rate, litter size, and live birth outcomes both under sustained hyperandrogenic conditions and following letrozole withdrawal under normalized androgen levels to determine if SF3B1 inhibition improves fertility outcomes in PMOS-like states. Moreover, given the strain-specific variations observed in PMOS susceptibility and systemic outcomes, it remains unknown whether SF3B1 inhibition yields similar benefits across other genetic backgrounds. Future studies using genetic models and extended treatment durations will offer more comprehensive insights into the chronic impact of SF3B1 inhibition.

While H3B-8800 has been tested in clinical trials for splice-resistant blood cancers (25, 26), its relevance to endocrine and metabolic disorders has not been previously explored. We observed minimal transcriptional effects in adipose depots; however, the effects of SF3B1 inhibition in other metabolically relevant tissues, including liver, muscle, and heart (99, 100), remain unclear. Given the central role of SF3B1 in RNA processing and its frequent mutations in hematologic malignancies (101), systemic SF3B1 inhibition may result in unintended risks in proliferative tissues. Investigating these tissue-specific effects will be essential for evaluating the therapeutic potential of SF3B1 splicing inhibition in PMOS. Similar to other complex diseases, limitations between mouse models and human disease should be carefully considered when drawing conclusions. Given the spectrum of physiologic changes observed in women with PMOS, no single model is sufficient to recapitulate the disease as a whole. Although rodent models offer high reproducibility and genetic tractability for investigating PMOS pathogenesis, a key limitation is their lack of menstruation (102). In rodents, reproductive health is typically assessed through estrous cyclicity, ovarian morphology (antral follicles, corpora lutea), and fertility metrics (litter size, pups per year), while in humans, menstrual cycle regularity is typically used as a clinical indicator of reproductive health. Notably, menstrual cycle irregularity has emerged as a potent predictor of cardiometabolic conditions (103), including obesity, diabetes, and cardiovascular diseases (104–106). Future studies that integrate estrous cycle assessments with uterine or endometrial endpoints in mouse models will be essential to better align with the menstrual cycle phenotypes observed in women with PMOS. Furthermore, defining how these measures track with menstruation using engineered models (107) will be a key first step in assessing the relevance of such comparisons.

Paradoxically, although continuous letrozole exposure in rodents induces PMOS-like phenotypes, short-term clinical use in patients can improve reproductive outcomes (27). This divergence likely reflects treatment timing: chronic aromatase inhibition in mice maintains hyperandrogenism and reproductive arrest, while brief, cycle-specific administration in humans reduces estrogen-mediated negative feedback, increases endogenous gonadotropin secretion, and promotes ovulation. Thus, continuous letrozole exposure models the consequences of sustained aromatase inhibition but does not recapitulate the treatment regimen or reproductive effects of clinical letrozole use. This distinction is an important limitation when translating findings from letrozole-induced rodent models to human PMOS, and it underscores the need to define which aspects of PMOS pathophysiology are conserved across species.

## Methods

### Sex as a biological variable.

Female mice were used in this study because PMOS is defined by ovarian dysfunction and hyperandrogenism-associated reproductive and cardiometabolic phenotypes. Publicly available proteomic data from male and female participants in the Atherosclerosis Risk in Communities (ARIC) study (65) were analyzed to assess sex-dependent differences.

### Animals.

Female inbred mouse strains were acquired from The Jackson Laboratory, and BXD strains were provided by the University of Tennessee Health Science Center. A complete list of strains, sources, and sample sizes is provided in Table 1. Mice were maintained under a 12-hour-light/dark cycle with ad libitum access to standard chow and water. All mice were housed in the same room under standardized conditions, including constant temperature, controlled ambient humidity, corncob bedding, and nestlet material. Mice received from The Jackson Laboratory or the University of Tennessee Health Science Center were allowed to acclimate for 2 weeks and underwent handling sessions before experiments. Female mice were 8–10 weeks of age at the start of treatment. All animal procedures were approved as described in the *Study approval* section.

### Letrozole-induced PMOS model.

The PMOS model was established using a protocol adapted from prior studies. Female mice, aged 8–10 weeks, received subcutaneous implants of letrozole or placebo pellets (3 mm diameter; Innovative Research of America) at the start of the study and again after 3 weeks to ensure continuous release of 50 μg/d letrozole over 6 weeks. Letrozole-treated and control mice were housed separately, with no more than 4 mice per cage. Mouse strains were selected based on availability from commercial or collaborative sources and to capture a range of hormonal and physiological variation that parallels heterogeneity observed in human PMOS cohorts. Clinical hormone ranges used to contextualize the mouse cohort are provided in Table 2.

### H3B-8800 treatment of PMOS mice.

PMOS mice underwent subcutaneous micro-osmotic pump implantation after 3 weeks of letrozole treatment, concurrent with the second letrozole pellet insertion. Micro-osmotic pumps (ALZET model 1004; DURECT Corporation; 100 μL reservoir; flow rate, 0.11 μL/h; 28-day delivery duration) were filled under sterile conditions with either H3B-8800 (2 mg/kg/day) or vehicle (1:4 v/v, 20% DMSO, 80% PEG 400) and primed for 48 hours at 37°C in sterile saline to ensure immediate drug delivery upon implantation. For subcutaneous implantation, mice were anesthetized with isoflurane in oxygen using 5% isoflurane for induction by chamber and 2%–3% isoflurane for maintenance by nose cone on a heated platform maintained at 37°C. Adequate anesthesia was assessed by absence of the toe-pinch reflex. The dorsal fur over the upper back was removed using depilatory cream, and the exposed skin was sterilized using povidone-iodine swabsticks. A small incision was made in the upper dorsal region using sterile surgical scissors. A subcutaneous pocket was created using sterile curved forceps. The pump was inserted with the flow moderator oriented posteriorly and downward to ensure continuous delivery. The incision was closed using sterile surgical Reflex 7 mm wound clips (CellPoint Scientific), and the site was treated with povidone-iodine. Mice were placed in a clean recovery cage and monitored until fully recovered from anesthesia. Mice were subsequently monitored daily throughout the postoperative period. Surgical clips were removed 7 days after implantation, and phenotypic assessments were performed after a 7-day recovery period. Unless otherwise indicated, comparisons between H3B-8800-treated and vehicle-treated mice were made at week 6, corresponding to 3 weeks after initiation of H3B-8800 or vehicle treatment and 6 weeks after the start of letrozole exposure.

### Body weight, body composition, and glucose tolerance testing.

Body weight was measured at the beginning of each week. Body composition was measured during week 5 of the protocol using the EchoMRI Whole Body Composition Analyzer (EchoMRI LLC). Intraperitoneal glucose tolerance tests were performed during week 5 in conscious mice. Briefly, mice were fasted for 6 hours and injected intraperitoneally with glucose at 1 g/kg body mass. Blood glucose levels were measured from the tail tip using a handheld glucometer (Accu-Chek Guide) at baseline and 15, 30, 45, 60, and 90 minutes after glucose administration.

### Echocardiographic analysis.

To assess cardiac structure and function, transthoracic echocardiography was performed during week 4 of the protocol using a VisualSonics Vevo 3100 system. Briefly, mice were anesthetized with 5% isoflurane, chest hair was removed, and mice were placed in a supine position on a heated platform with embedded ECG leads. Core temperature was maintained at 37°C. Anesthesia was maintained with 1%–3% isoflurane throughout the procedure. B-mode and M-mode images were acquired from parasternal short-axis and long-axis views to evaluate cardiac parameters, including left ventricular systolic and diastolic diameters, anterior and posterior wall thicknesses, left ventricular mass, fractional shortening, and ejection fraction. Using pulsed-wave Doppler, mean and peak velocities were measured in the ascending and descending aorta from the aortic arch.

### Estrous cycle assessment.

Owing to the number of in vivo assessments performed and to reduce stress on the animals, estrous cycle arrest, defined as constant diestrus for at least 2 complete cycles, was not assessed in the full cohort. Instead, 1 estrous cycle of up to 5 days was assessed during the final week of the protocol, week 6, which allowed determination of whether the last cycle followed normal stage progression, defined as diestrus-proestrus-estrus-metestrus, or was disrupted, defined as at least 3 days in the same stage. The estrous cycle stage was determined by light microscopic analysis of vaginal lavage smears. Proestrus was defined by the presence of mostly nucleated epithelial cells with some cornified epithelial cells, estrus by mostly cornified epithelial cells, metestrus by some cornified epithelial cells with mostly leukocytes, and diestrus by primarily leukocytes. For the H3B-8800 cohort, estrous cyclicity was assessed at 3 separate time points using the same criteria and procedures: 5 days during weeks 0–1 before letrozole treatment, 7 days during weeks 2–3 after letrozole treatment, and 7 days during weeks 5–6 after H3B-8800 or vehicle treatment.

### Tissue collection.

Physiological experiments, euthanasia, and implantations were performed within the same 3-hour window, from 1 to 4 PM during the light phase, to limit circadian variation. At the conclusion of the study, mice were euthanized using 2.5% isoflurane delivered by precision vaporizer followed by decapitation for fresh serum collection. Metabolic and reproductive tissues were collected, immediately frozen in liquid nitrogen, and stored at –80°C. To minimize technical variability, ovaries were preferentially allocated by side for downstream analyses, with the right ovary used for histological analysis and the left ovary used for RNA-seq. Ovaries designated for histology were fixed in 4% paraformaldehyde in phosphate-buffered saline at 4°C overnight, preserved in 70% ethanol, and processed for histology. Serum was collected after coagulation at room temperature for 1 hour by centrifugation at 2,000*g* for 10 minutes at 4°C and stored at –80°C for subsequent analyses.

### Ovarian morphology.

To estimate ovarian follicular populations, partial follicle counting and classification were performed in approximately one-quarter of each ovary per mouse in ovarian serial sections of 5 μm per section. Briefly, fixed ovarian tissue was serially sectioned, placed on gelatin-coated slides (Biobond, British Biocell International), air-dried for 2 hours, and fixed for 5 minutes in acetone at 4°C. Sections from each ovary were washed in PBS containing 137 mmol/L NaCl, 2.7 mmol/L KCl, 4.3 mmol/L Na_2_HPO_4_·7H_2_O, and 1.4 mmol/L KH2PO4, pH 7.3, and stained with hematoxylin and eosin (DAKO Corporation) for histological analysis. Serial sections were independently analyzed, and ovarian follicles were classified and quantified. The number and class of follicles were counted at regular intervals, as described, and the total number of follicles per ovary was estimated using a multiplication factor based on follicle class and sampling fraction. This estimation allowed quantification of primordial follicles; small follicles, including primary, secondary, and small antral follicles; and large follicles, including Graafian/preovulatory follicles. Ovarian cysts and corpora lutea were identified and counted as described previously. Follicular atresia was also quantified, and atretic follicles were defined as follicles with greater than 5% of granulosa cells containing pyknotic nuclei. Additional observations included hemorrhagic infiltration, increased vasculature, atretic debris, and lipid droplet-like structures.

### Serum hormonal levels.

Serum levels of luteinizing hormone, FSH, estradiol, and testosterone were assessed by the University of Virginia Center for Research in Reproduction Ligand Assay and Analysis Core. Serum insulin levels were measured using a Mouse Insulin ELISA (Alpco, catalog 80-INSMS-E01). For the H3B-8800 treatment cohort, serum testosterone levels were quantified using the Testosterone Parameter Assay Kit (R&D Systems, catalog KGE010).

### Bulk RNA-seq of ovarian and gonadal white adipose tissue.

Ovaries and gonadal white adipose tissue underwent total mRNA isolation using the Qiagen RNeasy micro kit with on-column DNA digestion according to the manufacturer’s protocol (QIAGEN, Rneasy Mini Kit, catalog 74104). For gonadal white adipose tissue, the entire gonadal fat pad surrounding the reproductive tract was collected and processed for RNA isolation. Library preparation was performed using the QuantSeq 3′ mRNA-Seq V2 Library Prep Kit with UDI to generate Illumina-compatible libraries, and sequencing was performed on the NovaSeq platform by Novogene. Transcript abundances were quantified from raw FASTQ files against the Mus musculus GRCm39 cDNA transcriptome using kallisto.GRCm39.cdna, using kallisto-aln. Version-specific Ensembl transcript IDs were linked to gene symbols using BioMart. Estimated counts were log-normalized and filtered for a minimum sum greater than 5 estimated counts across all samples. Differential expression analyses comparing PMOS and control samples across tissues were performed using limma. Differential expression results were visualized using R packages available through CRAN, including ggplot2, ggVennDiagram, and pheatmap. Gene set enrichment analyses and pathway networks of differentially expressed genes were performed using clusterProfiler.

### Variance partitioning analyses.

Mixed-effects models were used to estimate genetic and PMOS contributions to traits as shown previously. Briefly, variances were partitioned using a linear random slope model with treatment and strain as random effects. The proportion of total variance explained by strain was scaled to reflect a portion of residual variance from the previous model, representing a strain-by-treatment interaction. Linear modeling was performed using the lme function from the R package lme4, version 1.1-25. Variance plots were generated using the R package ggplot2, version 3.3.2.

### Cancer prediction signature analysis from ovarian trait genes.

Human orthologs for mouse genes were identified by intersecting mouse gene symbols with known human orthologs from the vertebrate homology resource at Mouse Genome Informatics. Sequencing and phenotype data for ovarian cancer from TCGA were accessed through the UCSC Xena portal (https://xena.ucsc.edu/) on December 21, 2024. Ovarian cancer data from the TCGA Ovarian Cancer (TCGA-OV) collection, which contains 489 patients, were used. Gene-level RNA-seq data in transcripts per million were analyzed for tumor versus surrounding tissue across the two pathways and compared using a Wilcoxon test. Survival assessments were performed by calculating the population mean for each gene in a given gene set, defined as cyst-correlated or ovarian area–correlated genes. Each patient was assigned a score based on the number of genes expressed above or below the mean. If half or more of the genes in each score were above the mean, the patient received a high score, whereas if fewer than half were above the mean, the patient received a low score. Survival curves were generated using the survminer and survfit packages in R, and statistical differences were assessed using a log-rank test.

### WGCNA analysis to define ovary-adipose crosstalk.

RNA-seq expression data from mouse ovary and adipose tissue were collapsed into modules using the WGCNA R package to identify clusters of correlated genes. Briefly, the goodSamplesGenes function from WGCNA was applied to identify and remove low-quality data with excessive missing values, although all samples passed initial quality control. Therefore, all genes used for differential expression were used for module construction. Blockwise module construction was then performed using the blockwiseModules function with a minimum module size of 200 genes, maximum module size of 2,000 genes, and standard merge cut height of 0.2. ME0 was removed because this module reflects genes that WGCNA could not assign to specific modules. Further integration with trait data allowed calculation of regression coefficients and corresponding *P* values between module eigengenes and traits using the bicorAndPvalue function from WGCNA. Correlations between module eigengenes and traits were used to construct undirected networks using qgraph.

### Human intertissue genetic correlations.

Human female expression data from GTEx were filtered to allow sufficient comparison of intertissue transcriptional correlation, as described previously. Correlations between ovarian genes and all human orthologs in target tissues were calculated using the bicorAndPvalue function in the WGCNA package. Pathway-specific genes corresponding to mitochondrial oxidation were obtained from Gene Ontology annotation for the combined terms “mitochondrion” and “oxidative phosphorylation.”

### Adipose single-cell sequencing integration.

Adipose single-cell sequencing data from Vijay et al. (44) were filtered to include only female samples and then rescaled and reclustered using Seurat with a resolution of 0.2. Cell annotations were assigned by combining enrichR annotations from Tabula Muris and Reactome with tissue compartment. From the resulting filtered matrix, human orthologs corresponding to intersected differentially expressed genes in PMOS were further analyzed for their aggregate correlation structure in the indicated cell types.

### Male versus female heart failure proteomics analysis.

Analyses focused on assessing differences between heart failure and control patients were performed using ARIC data (65). Proteomic data were first stratified by reported sex, and differential protein abundance was determined by comparing heart failure cases with noncases using a linear mixed model in limma. The model was adjusted for BMI, age, and diabetes status, and FDR adjustment was performed according to the number of proteins assayed.

### Alternative splicing analysis.

Alternative splicing events in response to H3B-8800 treatment were quantified using rMATS-turbo (73), version 4.3.0, with the *Mus musculus* genome assembly GRCm39/mm39 annotation. Junction counts were extracted, and 5 splicing event types were evaluated, including skipped exon, alternative 3′ splice site, alternative 5′ splice site, retained intron, and mutually exclusive exon events. Splicing events were processed in R using the maser package, version 1.20.0, and filtered for Δψ ≥ 10% and FDR ≤0.05. Splicing events passing these criteria were intersected with differentially expressed genes, defined as adjusted *P* < 0.05, between H3B-8800-treated and vehicle-treated mice for each tissue, including ovary, gonadal white adipose tissue, and subcutaneous white adipose tissue. For human KGN cells, alternative splicing was compared between DHT-treated and H3B-8800+DHT–treated cells using the same rMATS-turbo criteria.

### KGN cell culture.

Human KGN granulosa cells (Applied Biological Materials Inc., catalog T9195) were maintained in DMEM/F-12 medium supplemented with 10% fetal bovine serum and 1% v/v penicillin-streptomycin containing 100 U/mL penicillin and 100 μg/mL streptomycin at 37°C in a 5% CO_2_ incubator. Cells were seeded in 6-well plates at a density of 2 × 10^5^ cells per well and allowed to reach 80% confluency before treatment. DHT (1 mg/mL stock in DMSO; AbMole, catalog M6033) was diluted into culture medium to a final concentration of 100 nM. H3B-8800 (MedChemExpress, catalog HY-111517) was dissolved in DMSO and diluted in culture medium to a final concentration of 10 nM, with the final DMSO concentration maintained at 0.145% v/v across all treatment conditions. Experimental conditions included control, DHT, and H3B-8800 plus DHT, each performed in triplicate wells. After treatment, total RNA was isolated from KGN cells using TRIzol and a column-based purification kit with on-column DNase digestion according to the manufacturer’s protocol (RNeasy Mini Kit; QIAGEN, catalog 74104). Bulk RNA-seq was performed on KGN cells using the Illumina NovaSeq platform by Novogene to profile gene expression and alternative splicing changes.

### Statistics.

All computational procedures were performed using R statistical software. Correlations and associated *P* values were calculated using biweight midcorrelation in the WGCNA package, which is robust to outliers, with associated *P* values calculated using bicorAndPvalue. Pairwise comparisons between 2 groups were performed using 2-tailed Student’s *t* tests with 95% confidence intervals unless otherwise indicated. Survival analyses were assessed using log-rank tests. Differential expression analyses were performed as described above using limma, and FDR adjustment was applied where indicated. Values were considered statistically significant at *P* < 0.05. Where error bars are present, data are shown as mean ± SEM.

### Study approval.

All animal procedures were reviewed and approved by the Institutional Animal Care and Use Committee of the University of California, Irvine, under protocol AUP-22-102. All animal studies were conducted in accordance with institutional guidelines for the care and use of laboratory animals. Publicly available or previously published deidentified human datasets were analyzed as secondary data; therefore, additional institutional review board approval and informed consent were not required for the present analyses.

### Data availability.

All data from the genetic screen have been deposited into GeneNetwork and are located under the HMDP and BXD families of datasets, where analytical tools are available to study complex networks of genes and phenotypes. RNA-seq data generated from the genetic diversity screen and H3B-8800 tissue profiling have been deposited in the NCBI Sequence Read Archive under accession PRJNA1250774. Supporting data values associated with the main text and supplemental material, including values for all data points shown in graphs and values behind reported means, are provided in the Supporting Data Values file.

## Author contributions

CMN and LMV collected data and contributed to experimental design, conceptualization, interpretation of results, and drafting and editing the manuscript. Author order was determined by corresponding authors during two formal meetings where relative contributions of each author were discussed. YC and IM provided insight and assistance with respect to repurposing spliceosome inhibitors. CLJ, CDJ, IT, MZ, EA, IY, FD, ML, TG, KO, NM, DK, CHV, NU, NRP, SM, EZK, GM, VS, CX, and MB provided expertise and experimental assistance with respect to mouse and human studies. AS, MEN, and DEJ provided assistance on heritability estimates and variance partitioning. ASK helped develop the mouse model for PMOS and validated reproducibility across genetic backgrounds. CJ facilitated metabolic studies of organ crosstalk. RWW and DGA provided the BXD strains and, together with EGW, provided critical insight regarding mouse genetic analyses AS, MEN, and DEJ performed heritability estimates and contributed insights with regard to network-based views. IM, DN, and MS oversaw experimental design, implementation, drafting and editing, and interpretation of results. All authors have read and reviewed the submitted manuscript.

## Conflict of interest

The authors have declared that no conflict of interest exists.

## Funding support

This work is the result of NIH funding, in whole or in part, and is subject to the NIH Public Access Policy. Through acceptance of this federal funding, the NIH has been given a right to make the work publicly available in PubMed Central.

- NIH grants U54OD039864, R01HD111650, R00HL138193, T34GM136498, and DP1DK130640.

## Supplementary Material

Supplemental data

Supplemental table 1

Supplemental table 2

Supplemental table 3

Supplemental table 4

Supplemental table 5

Supplemental table 6

Supplemental table 7

Supporting data values

## Figures and Tables

**Table 1:**
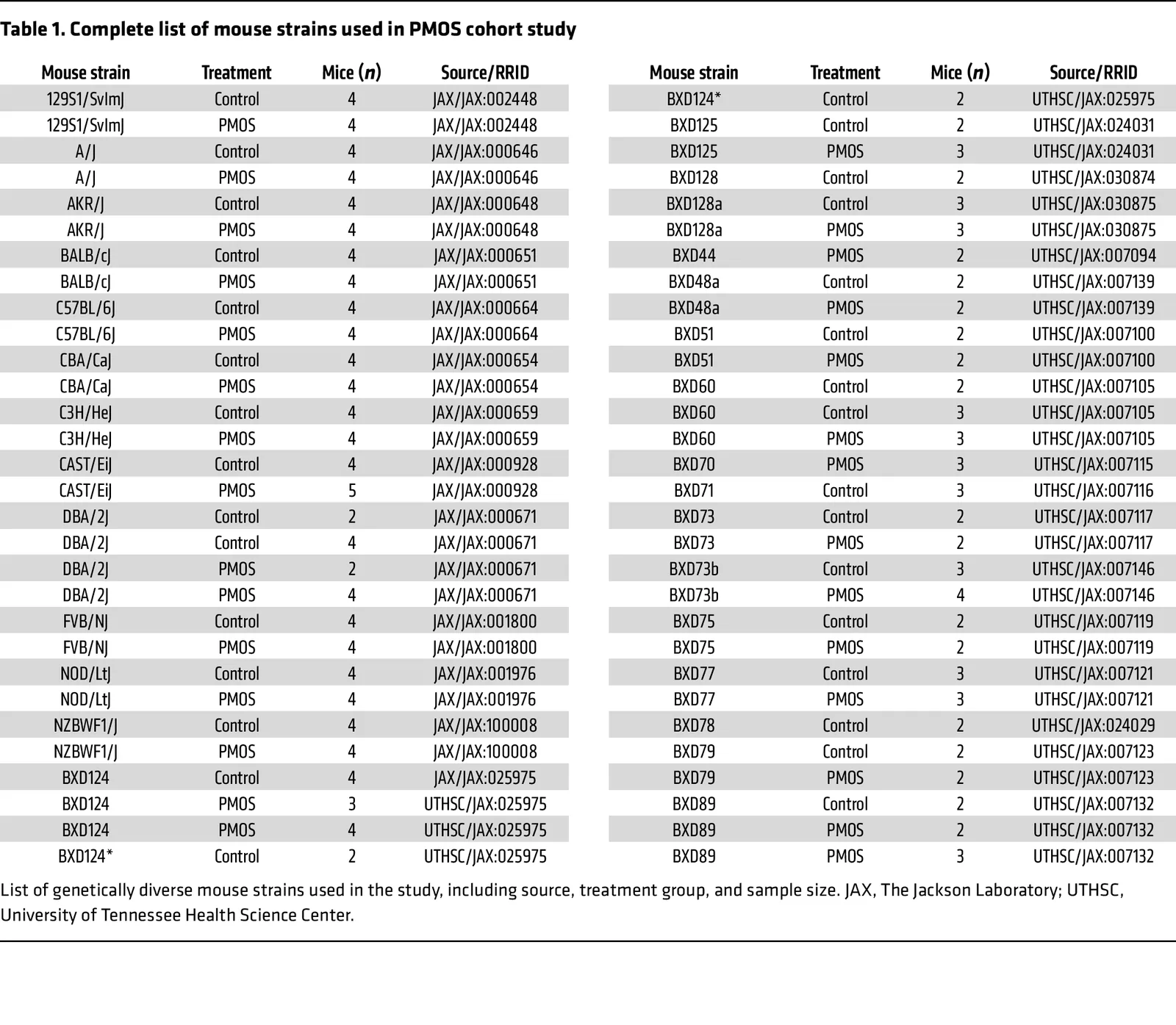
Complete list of mouse strains used in PMOS cohort study

**Table 2:**
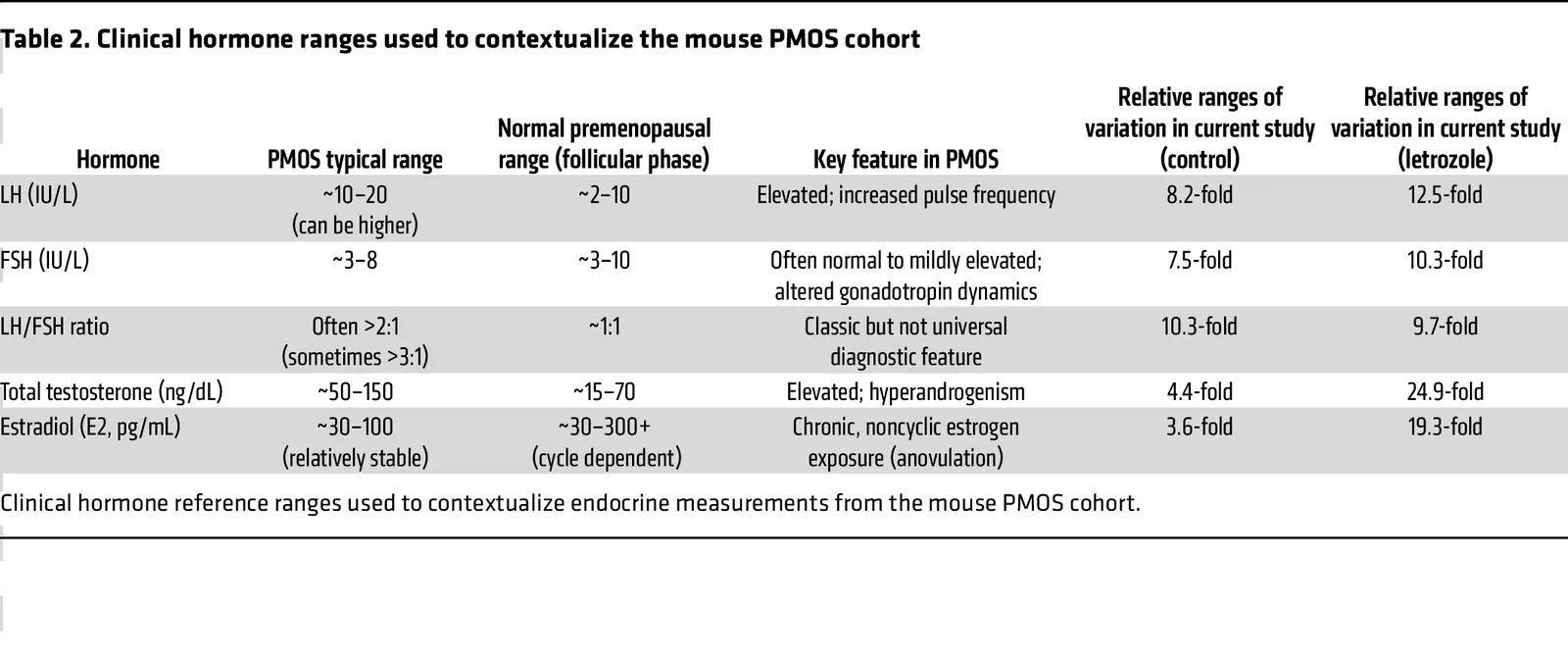
Clinical hormone ranges used to contextualize the mouse PMOS cohort

## Version 1 08/06/2026

In-Press Preview

## Version 2 09/15/2026

Electronic publication
