# Supplementary material for: Systems genetics approaches model the heritable architecture of polyendocrine metabolic ovarian syndrome
Source: J Clin Invest. 2026 Aug 6;136(18):e198215. doi: 10.1172/JCI198215 (PMC13574150; doi:10.1172/JCI198215)
Supplement: Supplementary file 1 [file jci-136-198215-s067.pdf]

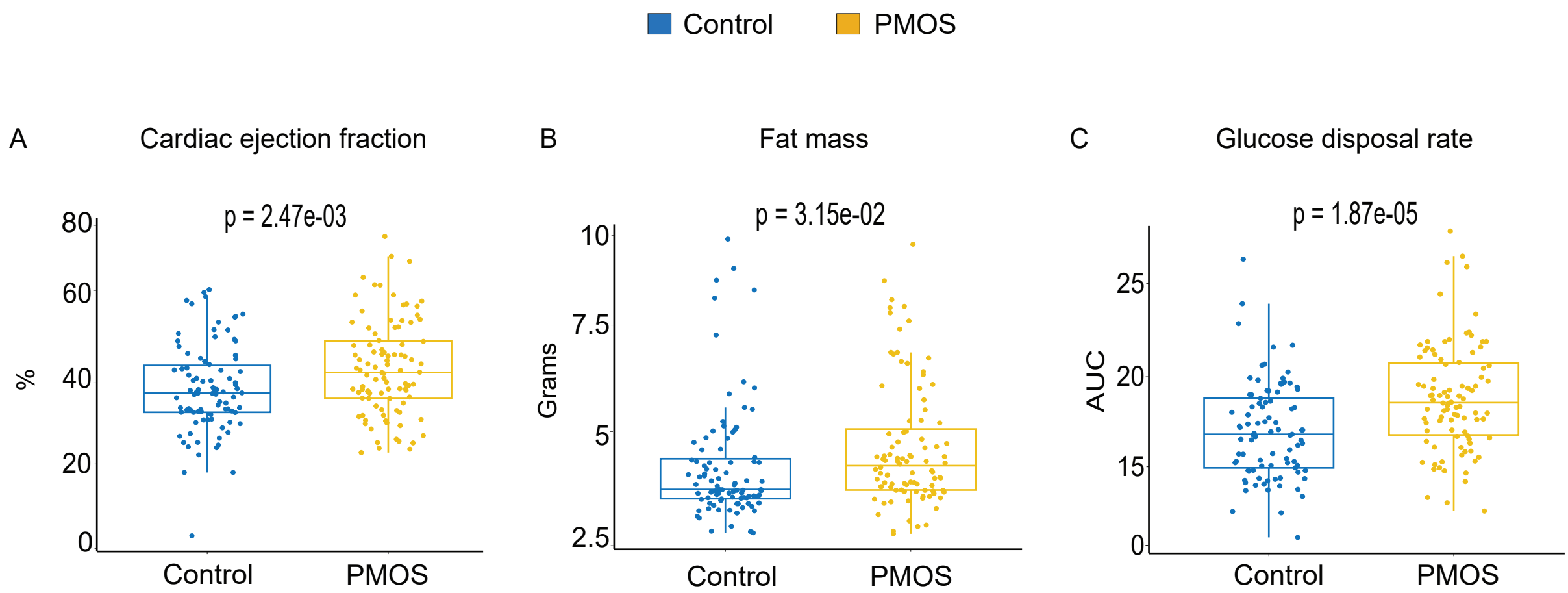

Control
PMOS
A
Cardiac ejection fraction
p = 2.47e-03
%
80
60
40
20
0
Control
PMOS
B
Fat mass
p = 3.15e-02
Grams
10
7.5
5
2.5
Control
PMOS
C
Glucose disposal rate
p = 1.87e-05
AUC
25
20
15
0
Control
PMOS

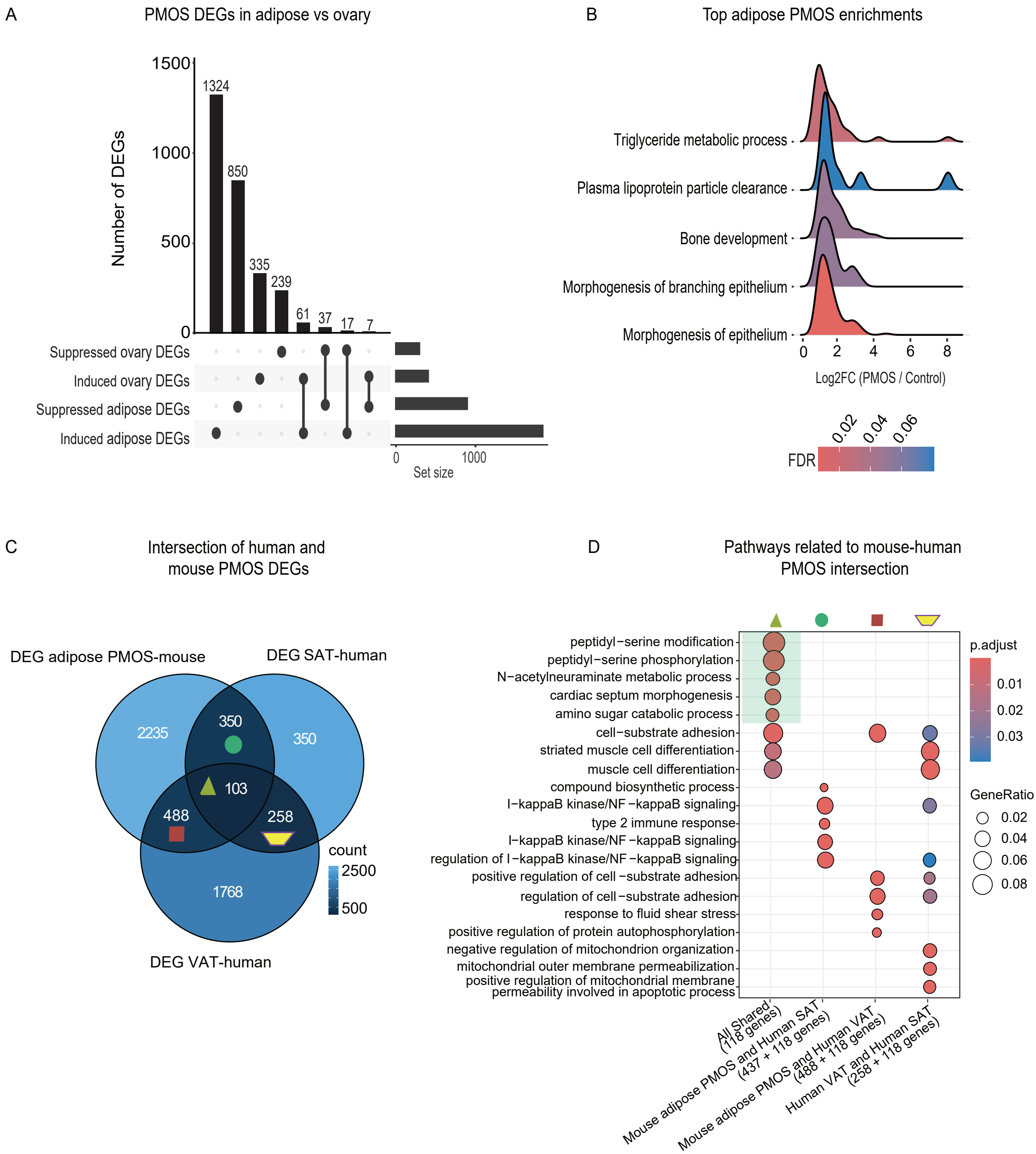

A
PMOS DEGs in adipose vs ovary
Number of DEGs
1500
1000
500
0
1324
850
335
239
61
37
17
7
Suppressed ovary DEGs
Induced ovary DEGs
Suppressed adipose DEGs
Induced adipose DEGs
0
1000
Set size
B
Top adipose PMOS enrichments
Triglyceride metabolic process
Plasma lipoprotein particle clearance
Bone development
Morphogenesis of branching epithelium
Morphogenesis of epithelium
0
2
4
6
8
Log2FC (PMOS / Control)
FDR
0.02
0.04
0.06
C
Intersection of human and mouse PMOS DEGs
DEG adipose PMOS-mouse
DEG SAT-human
DEG VAT-human
2235
350
350
103
488
258
1768
count
2500
500
D
Pathways related to mouse-human PMOS intersection
peptidyl−serine modification
peptidyl−serine phosphorylation
N−acetylneuraminate metabolic process
cardiac septum morphogenesis
amino sugar catabolic process
cell−substrate adhesion
striated muscle cell differentiation
muscle cell differentiation
compound biosynthetic process
I−kappaB kinase/NF−kappaB signaling
type 2 immune response
I−kappaB kinase/NF−kappaB signaling
regulation of I−kappaB kinase/NF−kappaB signaling
positive regulation of cell−substrate adhesion
regulation of cell−substrate adhesion
response to fluid shear stress
positive regulation of protein autophosphorylation
negative regulation of mitochondrion organization
mitochondrial outer membrane permeabilization
positive regulation of mitochondrial membrane permeability involved in apoptotic process
All Shared (118 genes)
Mouse adipose PMOS and Human SAT (437 + 118 genes)
Mouse adipose PMOS and Human VAT (488 + 118 genes)
Human VAT and Human SAT (258 + 118 genes)
p.adjust
0.01
0.02
0.03
GeneRatio
0.02
0.04
0.06
0.08

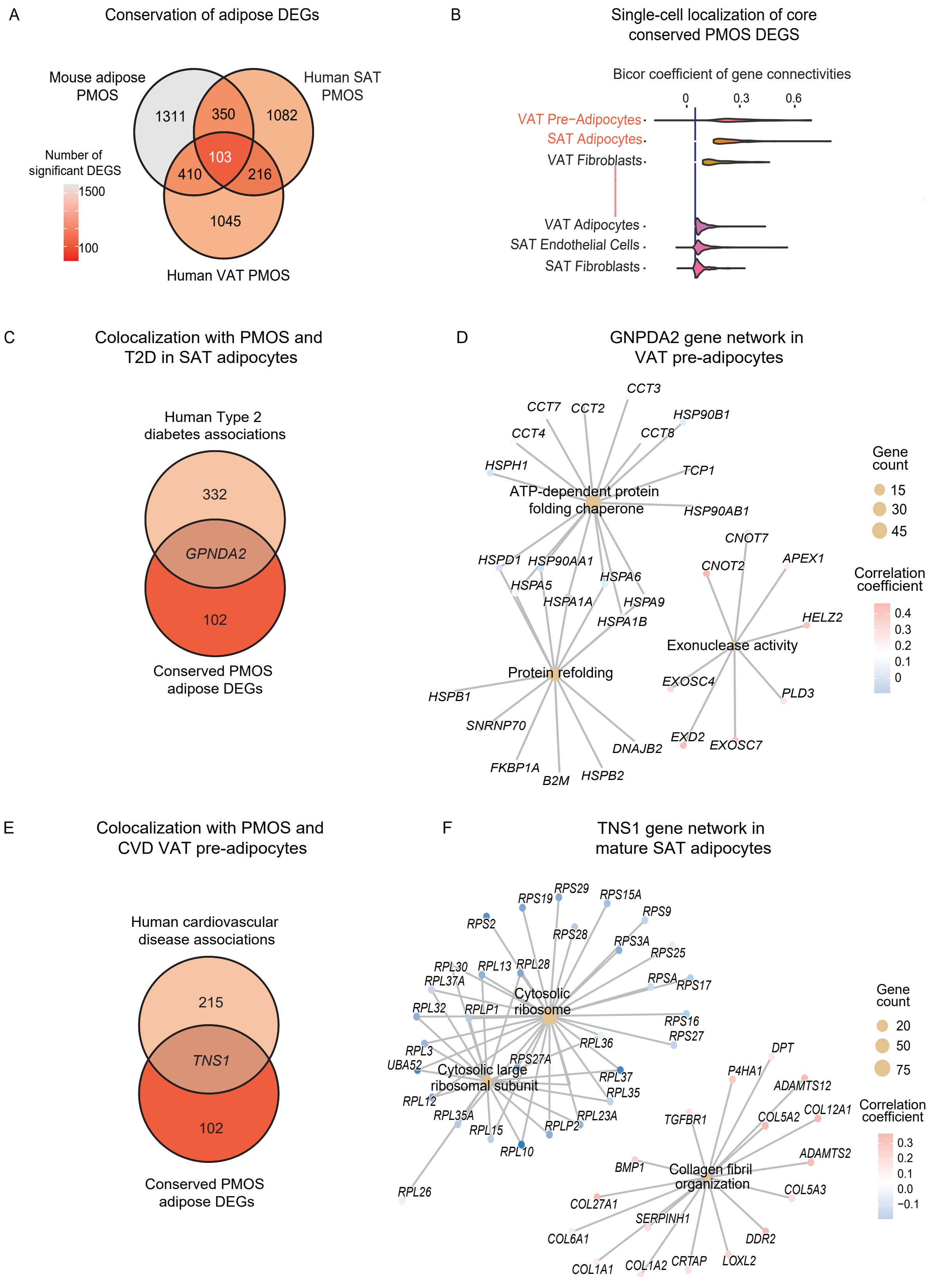

A
Conservation of adipose DEGs
Mouse adipose PMOS
Human SAT PMOS
Human VAT PMOS
1311
350
1082
103
410
216
1045
Number of significant DEGS
1500
100
B
Single-cell localization of core conserved PMOS DEGS
Bicor coefficient of gene connectivities
0
0.3
0.6
VAT Pre−Adipocytes
SAT Adipocytes
VAT Fibroblasts
VAT Adipocytes
SAT Endothelial Cells
SAT Fibroblasts
C
Colocalization with PMOS and T2D in SAT adipocytes
Human Type 2 diabetes associations
332
GPNDA2
102
Conserved PMOS adipose DEGs
D
GNPDA2 gene network in VAT pre-adipocytes
CCT3
CCT7
CCT2
HSP90B1
CCT4
CCT8
HSPH1
TCP1
ATP-dependent protein folding chaperone
HSP90AB1
CNOT7
HSPD1
HSP90AA1
CNOT2
APEX1
HSPA6
HSPA5
HSPA1A
HSPA9
HELZ2
HSPA1B
Exonuclease activity
Protein refolding
EXOSC4
HSPB1
PLD3
SNRNP70
EXD2
EXOSC7
DNAJB2
FKBP1A
B2M
HSPB2
Gene count
15
30
45
Correlation coefficient
0.4
0.3
0.2
0.1
0
E
Colocalization with PMOS and CVD VAT pre-adipocytes
Human cardiovascular disease associations
215
TNS1
102
Conserved PMOS adipose DEGs
F
TNS1 gene network in mature SAT adipocytes
RPS29
RPS19
RPS15A
RPS9
RPS2
RPS28
RPS3A
RPS25
RPL30
RPL13
RPL28
RPL37A
RPSA
RPS17
Cytosolic ribosome
RPL32
RPLP1
RPS16
RPS27
RPL36
RPL3
UBA52
RPS27A
Cytosolic large ribosomal subunit
RPL37
RPL35
RPL12
RPL35A
RPL23A
RPLP2
RPL15
RPL10
RPL26
DPT
P4HA1
ADAMTS12
COL12A1
COL5A2
TGFBR1
ADAMTS2
BMP1
Collagen fibril organization
COL5A3
COL27A1
SERPINH1
COL6A1
DDR2
COL1A1
COL1A2
CRTAP
LOXL2
Gene count
20
50
75
Correlation coefficient
0.3
0.2
0.1
0.0
−0.1

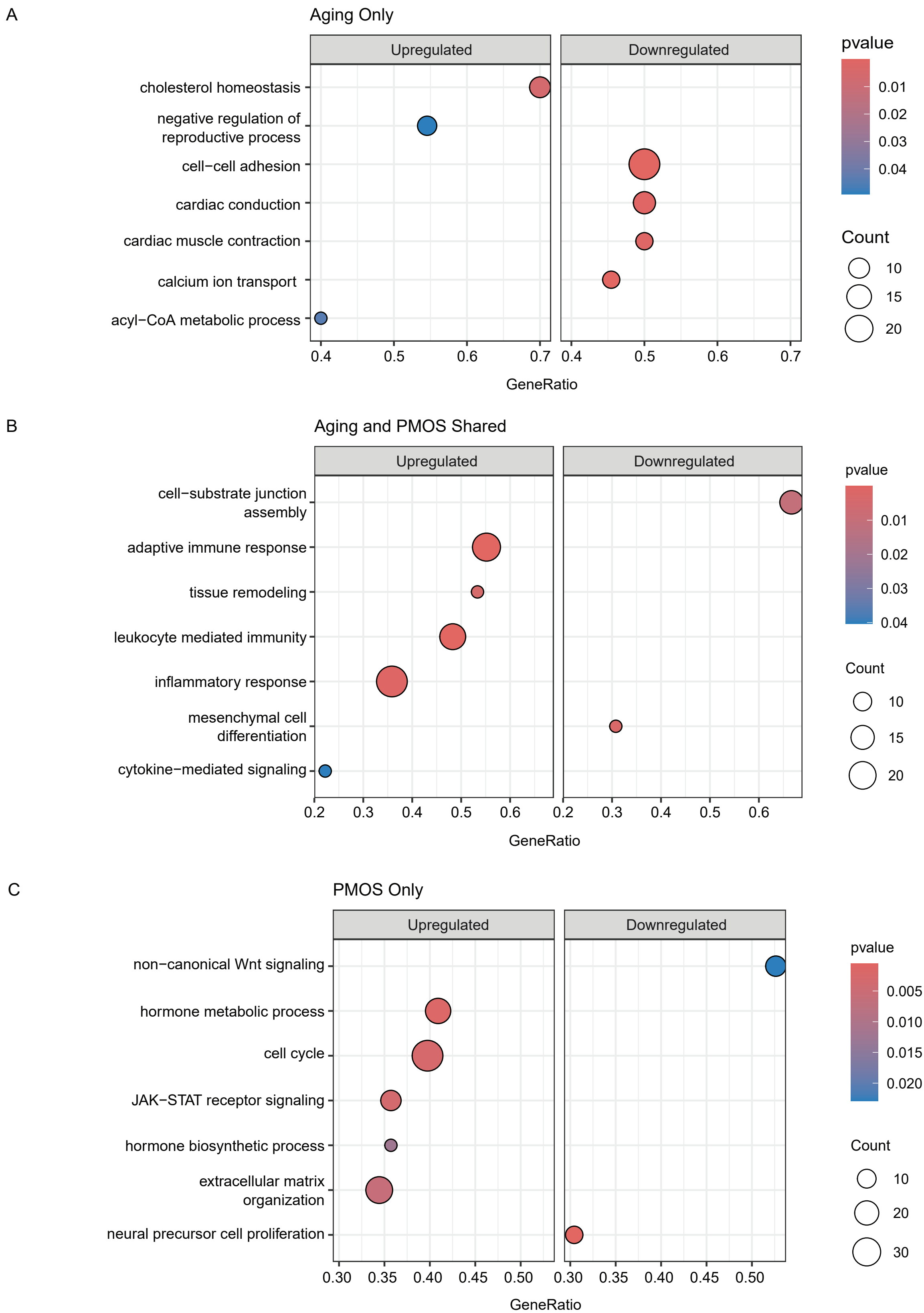
A
Aging Only
Upregulated
Downregulated
cholesterol homeostasis
negative regulation of reproductive process
cell−cell adhesion
cardiac conduction
cardiac muscle contraction
calcium ion transport
acyl−CoA metabolic process
0.4
0.5
0.6
0.7
GeneRatio
pvalue
0.01
0.02
0.03
0.04
Count
10
15
20
B
Aging and PMOS Shared
Upregulated
Downregulated
cell−substrate junction assembly
adaptive immune response
tissue remodeling
leukocyte mediated immunity
inflammatory response
mesenchymal cell differentiation
cytokine−mediated signaling
0.2
0.3
0.4
0.5
0.6
GeneRatio
pvalue
0.01
0.02
0.03
0.04
Count
10
15
20
C
PMOS Only
Upregulated
Downregulated
non−canonical Wnt signaling
hormone metabolic process
cell cycle
JAK−STAT receptor signaling
hormone biosynthetic process
extracellular matrix organization
neural precursor cell proliferation
0.30
0.35
0.40
0.45
0.50
GeneRatio
pvalue
0.005
0.010
0.015
0.020
Count
10
20
30

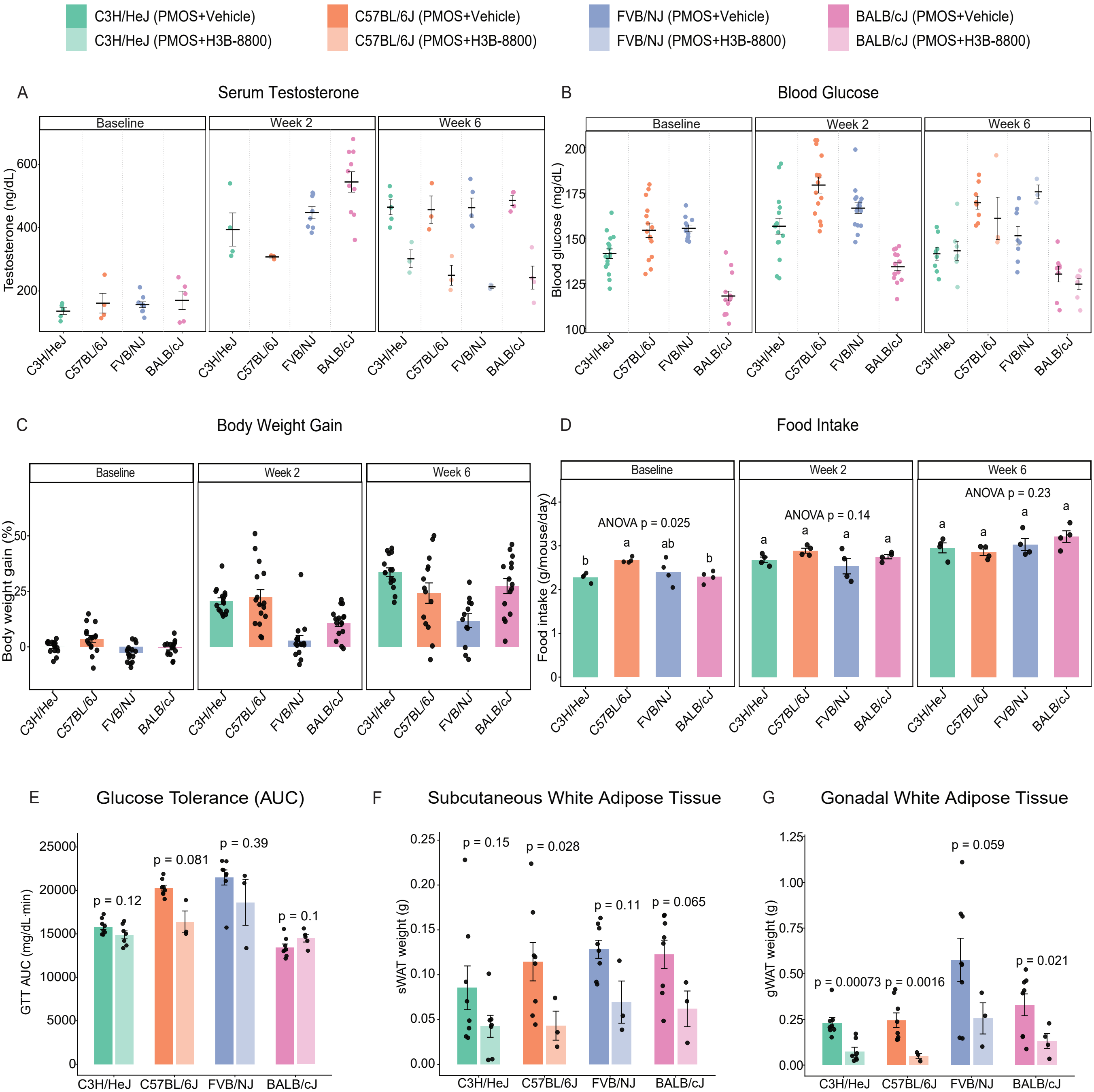

C3H/HeJ (PMOS+Vehicle)
C3H/HeJ (PMOS+H3B-8800)
C57BL/6J (PMOS+Vehicle)
C57BL/6J (PMOS+H3B-8800)
FVB/NJ (PMOS+Vehicle)
FVB/NJ (PMOS+H3B-8800)
BALB/cJ (PMOS+Vehicle)
BALB/cJ (PMOS+H3B-8800)
A
Serum Testosterone
Baseline
Week 2
Week 6
Testosterone (ng/dL)
B
Blood Glucose
Blood glucose (mg/dL)
C
Body Weight Gain
Body weight gain (%)
D
Food Intake
Food intake (g/mouse/day)
ANOVA p = 0.025
ANOVA p = 0.14
ANOVA p = 0.23
C3H/HeJ
C57BL/6J
FVB/NJ
BALB/cJ
E
Glucose Tolerance (AUC)
GTT AUC (mg/dL·min)
p = 0.12
p = 0.081
p = 0.39
p = 0.1
F
Subcutaneous White Adipose Tissue
sWAT weight (g)
p = 0.15
p = 0.028
p = 0.11
p = 0.065
G
Gonadal White Adipose Tissue
gWAT weight (g)
p = 0.00073
p = 0.0016
p = 0.059
p = 0.021

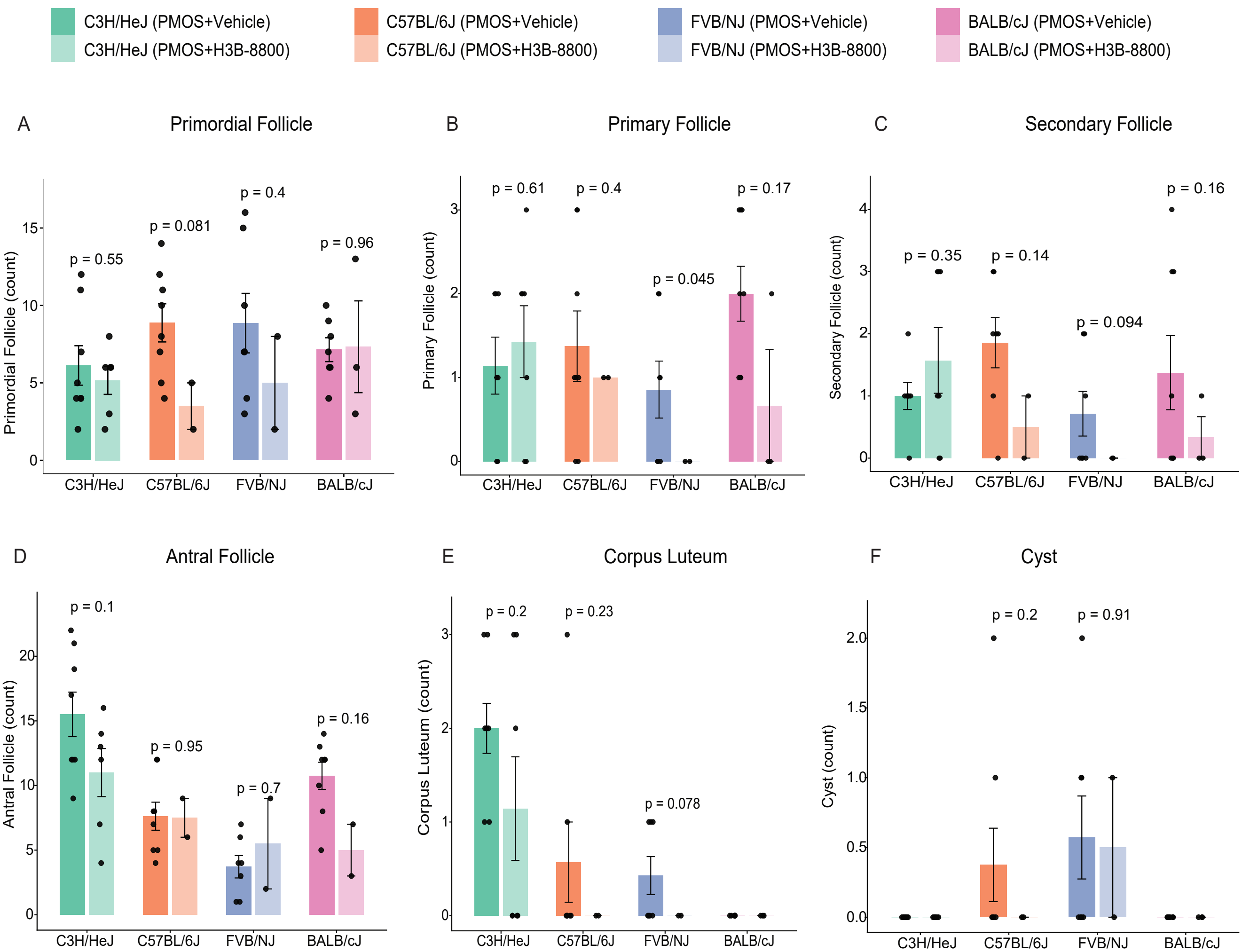

C3H/HeJ (PMOS+Vehicle)
C3H/HeJ (PMOS+H3B-8800)
C57BL/6J (PMOS+Vehicle)
C57BL/6J (PMOS+H3B-8800)
FVB/NJ (PMOS+Vehicle)
FVB/NJ (PMOS+H3B-8800)
BALB/cJ (PMOS+Vehicle)
BALB/cJ (PMOS+H3B-8800)
A
Primordial Follicle
Primordial Follicle (count)
p = 0.55
p = 0.081
p = 0.4
p = 0.96
B
Primary Follicle
Primary Follicle (count)
p = 0.61
p = 0.4
p = 0.045
p = 0.17
C
Secondary Follicle
Secondary Follicle (count)
p = 0.35
p = 0.14
p = 0.094
p = 0.16
D
Antral Follicle
Antral Follicle (count)
p = 0.1
p = 0.95
p = 0.7
p = 0.16
E
Corpus Luteum
Corpus Luteum (count)
p = 0.2
p = 0.23
p = 0.078
F
Cyst
Cyst (count)
p = 0.2
p = 0.91
C3H/HeJ
C57BL/6J
FVB/NJ
BALB/cJ

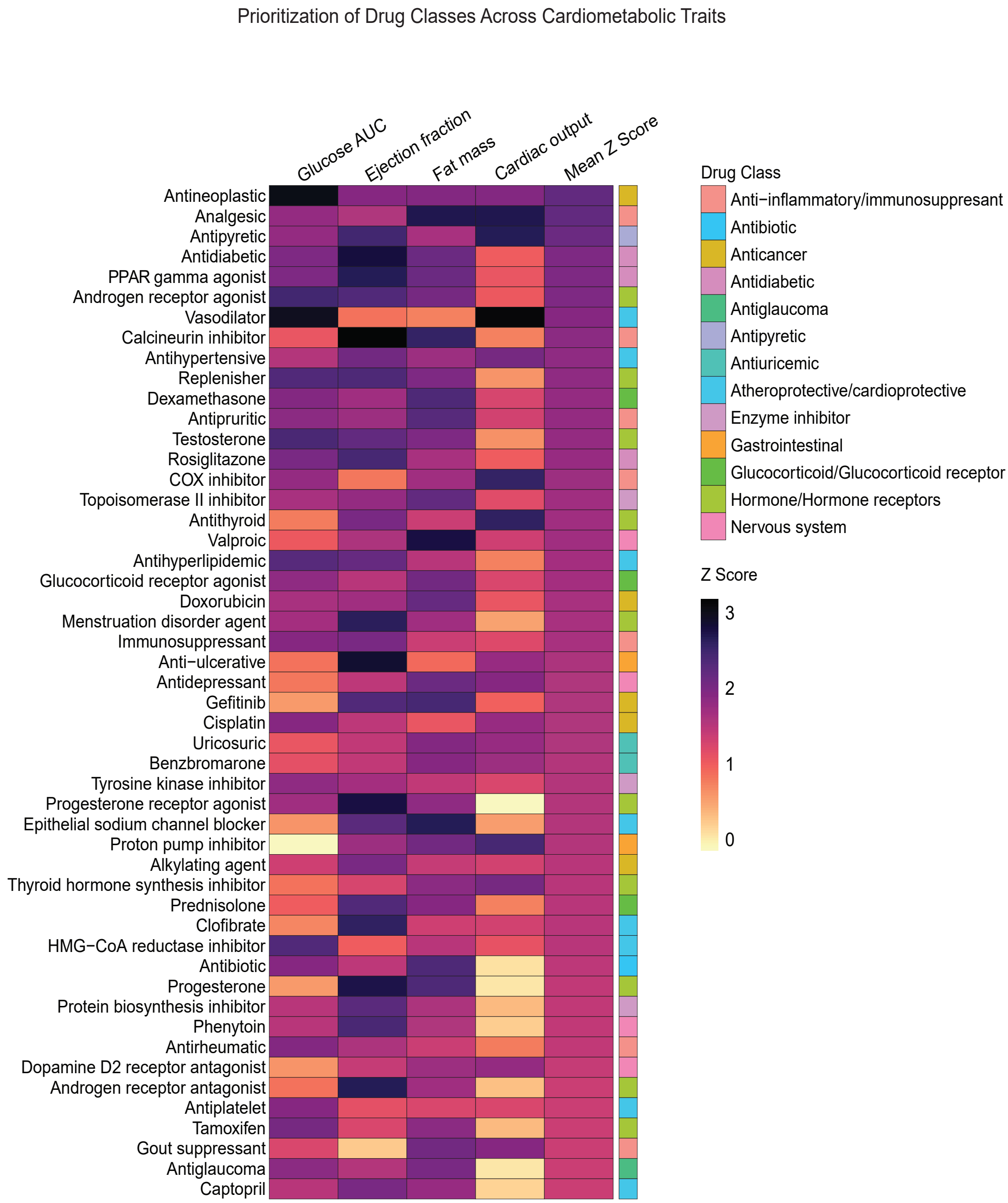

Prioritization of Drug Classes Across Cardiometabolic Traits
Glucose AUC
Ejection fraction
Fat mass
Cardiac output
Mean Z Score
Antineoplastic
Analgesic
Antipyretic
Antidiabetic
PPAR gamma agonist
Androgen receptor agonist
Vasodilator
Calcineurin inhibitor
Antihypertensive
Replenisher
Dexamethasone
Antipruritic
Testosterone
Rosiglitazone
COX inhibitor
Topoisomerase II inhibitor
Antithyroid
Valproic
Antihyperlipidemic
Glucocorticoid receptor agonist
Doxorubicin
Menstruation disorder agent
Immunosuppressant
Anti−ulcerative
Antidepressant
Gefitinib
Cisplatin
Uricosuric
Benzbromarone
Tyrosine kinase inhibitor
Progesterone receptor agonist
Epithelial sodium channel blocker
Proton pump inhibitor
Alkylating agent
Thyroid hormone synthesis inhibitor
Prednisolone
Clofibrate
HMG−CoA reductase inhibitor
Antibiotic
Progesterone
Protein biosynthesis inhibitor
Phenytoin
Antirheumatic
Dopamine D2 receptor antagonist
Androgen receptor antagonist
Antiplatelet
Tamoxifen
Gout suppressant
Antiglaucoma
Captopril
Drug Class
Anti−inflammatory/immunosuppresant
Antibiotic
Anticancer
Antidiabetic
Antiglaucoma
Antipyretic
Antiuricemic
Atheroprotective/cardioprotective
Enzyme inhibitor
Gastrointestinal
Glucocorticoid/Glucocorticoid receptor
Hormone/Hormone receptors
Nervous system
Z Score
3
2
1
0

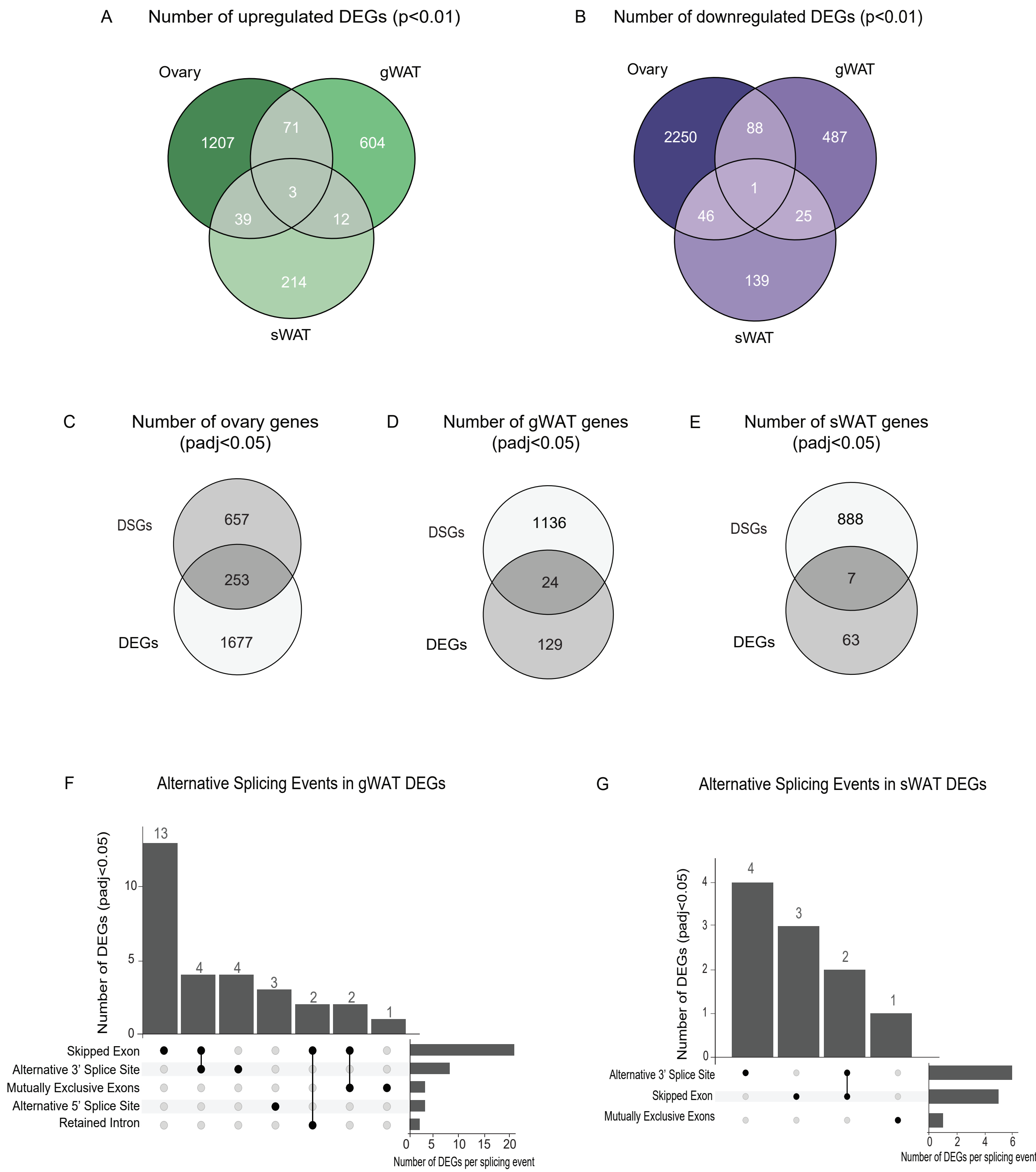

A
Number of upregulated DEGs (p<0.01)
Ovary
gWAT
sWAT
1207
71
604
3
39
12
214
B
Number of downregulated DEGs (p<0.01)
Ovary
gWAT
sWAT
2250
88
487
1
46
25
139
C
Number of ovary genes (padj<0.05)
DSGs
DEGs
657
253
1677
D
Number of gWAT genes (padj<0.05)
DSGs
DEGs
1136
24
129
E
Number of sWAT genes (padj<0.05)
DSGs
DEGs
888
7
63
F
Alternative Splicing Events in gWAT DEGs
Number of DEGs (padj<0.05)
13
4
4
3
2
2
1
Skipped Exon
Alternative 3' Splice Site
Mutually Exclusive Exons
Alternative 5' Splice Site
Retained Intron
Number of DEGs per splicing event
G
Alternative Splicing Events in sWAT DEGs
Number of DEGs (padj<0.05)
4
3
2
1
Alternative 3' Splice Site
Skipped Exon
Mutually Exclusive Exons
Number of DEGs per splicing event

Supplemental Figure 1. Global PMOS induction of metabolic traits. A-C, Cardiac ejection fraction (A), fat mass (B), and glucose disposal rate, measured as area under the curve during glucose tolerance testing (C), in control and letrozole-induced PMOS mice. Points represent individual mice; boxes show the median and interquartile range, and whiskers extend to 1.5× the interquartile range. P values were determined by unpaired 2-tailed Student's t test.

Supplemental Figure 2. Conserved depot-specific adipose landscape of PMOS. A, Number of differentially expressed genes in ovary and adipose tissue from PMOS mice compared with control mice. Bars indicate genes suppressed or induced in each tissue. B, Top enriched pathways among adipose PMOS-associated differentially expressed genes, shown by log2 fold change in PMOS relative to control mice. Color indicates FDR. C, Intersection of differentially expressed genes between mouse PMOS gonadal adipose tissue and human PMOS subcutaneous adipose tissue or visceral adipose tissue. D, Gene Ontology overrepresentation analysis of gene sets identified from the intersections shown in C. Dot size indicates gene ratio, and color indicates adjusted P value.

Supplemental Figure 3. Integration of conserved PMOS adipose genes with single-cell and genome-wide association datasets prioritizes molecular links between PMOS, cardiovascular disease, and type 2 diabetes. A, Overlap of PMOS-associated adipose differentially expressed genes across mouse adipose tissue and human subcutaneous or visceral adipose tissue. B, Single-cell localization of core conserved PMOS adipose differentially expressed genes across female adipose cell populations. C, Colocalization of conserved PMOS adipose genes with human type 2 diabetes-associated genes. D, Gene network centered on *GNPDA2* in visceral adipose pre-adipocytes, highlighting connected pathways related to protein folding and exoribonuclease activity. E, Colocalization of conserved PMOS adipose genes with human cardiovascular disease-associated genes. F, Gene network centered on *TNS1* in mature subcutaneous adipocytes, highlighting connected pathways related to ribosomal and extracellular matrix remodeling. Gene network node size indicates gene count, and color indicates correlation coefficient.

Supplemental Figure 4. Gene set enrichment analysis of ovarian transcriptomic profiles reveals distinct and shared biological processes in PMOS and aging. A-C, Gene Ontology enrichment analysis of ovarian transcriptomic signatures showing biological processes associated with aging-specific genes (A), genes shared between aging and PMOS (B), and PMOS-specific genes (C). Terms are separated by upregulated and downregulated gene sets. Dot size indicates gene count, dot color indicates P value, and the x-axis shows gene ratio.

Supplemental Figure 5. Hormonal and metabolic phenotyping across genetically diverse PMOS mice treated with H3B-8800. A and B, Serum testosterone (A) and blood glucose (B) measured at baseline, week 2, and the vehicle/H3B-8800 comparison time point in letrozole-induced PMOS mice across C3H/HeJ, C57BL/6J, FVB/NJ, and BALB/cJ strains. Baseline and week 2 measurements were collected before H3B-8800 treatment initiation and are shown by strain to reflect responses to letrozole exposure; the final time point is shown by treatment group to compare PMOS + vehicle and PMOS + H3B-8800 mice. C, Body weight gain, calculated as percent change from baseline, across baseline, week 2, and the final treatment time point. D, Daily food intake measured across the same time points. E, Glucose tolerance test area under the curve (GTT AUC). F and G, Subcutaneous white adipose tissue (sWAT) weight (F) and gonadal white adipose tissue (gWAT) weight (G). Where treatment groups are shown, dark colors indicate PMOS + vehicle and light colors indicate PMOS + H3B-8800 within each strain: C3H/HeJ, green; C57BL/6J, orange; FVB/NJ, blue; and BALB/cJ, pink. Vehicle and H3B-8800 comparisons were performed 3 weeks after initiation of vehicle or H3B-8800 treatment, following 3 weeks of letrozole-induced PMOS induction. Points represent individual mice; bars show mean ± SEM. Sample sizes vary by assay and are indicated by individual data points, with n = 3–8 mice per strain per treatment group. For D, ANOVA P values are shown above each time point, and different letters indicate significant strain differences by post hoc multiple-

comparison testing. For E–G, P values were determined by unpaired two-tailed Student's t tests comparing vehicle and H3B-8800 treatment within each strain.

Supplemental Figure 6. Ovarian morphology across genetically diverse mouse models of PMOS treated with H3B-8800. A, Primordial follicle, B, primary follicle, C, secondary follicle, D, antral follicle, E, corpus luteum, and F, cyst counts across C3H/HeJ (green), C57BL/6J (orange), FVB/NJ (blue), and BALB/cJ (pink) mice treated with vehicle (dark color) or H3B-8800 (light color) under letrozole-induced PMOS-like conditions (n = 2-8 mice per strain per treatment group). Data are shown as mean ± SEM. Statistical comparisons were performed using unpaired two-tailed t-tests within each strain.

Supplemental Figure 7. Compound enrichment scores for PMOS trait-correlated ovarian gene networks. Z-score heatmap showing prioritization of drug classes based on network-based enrichment of drug-induced transcriptomic signatures relative to ovarian gene sets associated with PMOS-related cardiometabolic traits, including glucose area under the curve, ejection fraction, fat mass, and cardiac output. The mean Z score summarizes enrichment across traits. Drug class annotations are shown on the right. Enrichment scores were computed using the PharmOmics platform.

Supplemental Figure 8. Tissue-specific transcriptional and splicing alterations in PMOS mice treated with H3B-8800. A and B, Overlap of upregulated (A) and downregulated (B) differentially expressed genes across ovary, gonadal white adipose tissue, and subcutaneous white adipose tissue in PMOS mice treated with H3B-8800 versus vehicle. C-E, Overlap between differentially spliced genes and differentially expressed genes in ovary (C), gonadal white adipose tissue (D), and subcutaneous white adipose tissue (E). F and G, Alternative splicing event types among differentially expressed genes in gonadal white adipose tissue (F) and subcutaneous white adipose tissue (G). Bar heights indicate the number of differentially expressed genes associated with each splicing event combination, and the horizontal bars indicate the total number of genes per splicing event type. Differential expression was assessed using limma, and alternative splicing events were identified using rMATS.

Table Legends

Supplemental Table 1. Phenotypic traits measured in the mouse study. Summary of the metabolic, cardiac, hormonal, and reproductive traits measured across the genetically diverse mouse cohort for each strain.

Supplemental Table 2. Ovarian RNA-seq differential expression analysis comparing PMOS and control mice. Differential expression results from ovarian RNA-seq comparing PMOS versus control mice.

Supplemental Table 3. Marker genes used for ovarian single-cell deconvolution. Cell-type marker genes used to estimate ovarian cell-type-specific transcriptional signatures.

Supplemental Table 4. Comparison of ovarian cancer prediction models. Comparison of cancer prediction robustness between the model developed from ovarian size variation and previously published models developed to predict ovarian cancer outcomes. Study references are shown as PubMed IDs.

Supplemental Table 5. Adipose RNA-seq differential expression analysis comparing PMOS and control mice. Differential expression results from adipose tissue RNA-seq comparing PMOS versus control mice.

Supplemental Table 6. RNA-seq differential expression analysis comparing H3B-8800 and vehicle treatment. Differential expression results from ovary and adipose tissues comparing H3B-8800-treated versus vehicle-treated PMOS mice.

Supplemental Table 7. KGN cell RNA-seq differential expression analysis comparing DHT and control treatment. Differential expression results from human KGN granulosa cells comparing DHT-treated versus control conditions.
